# Nucleation and spreading of a heterochromatic domain in fission yeast

**DOI:** 10.1038/ncomms11518

**Published:** 2016-05-11

**Authors:** Michaela J. Obersriebnig, Emil M. H. Pallesen, Kim Sneppen, Ala Trusina, Geneviève Thon

**Affiliations:** 1 Department of Biology, University of Copenhagen, BioCenter, Ole Maaløes Vej 5, 2200 Copenhagen, Denmark; 2 Niels Bohr Institute, University of Copenhagen, Blegdamsvej 17, 2100 Copenhagen, Denmark

## Abstract

Outstanding questions in the chromatin field bear on how large heterochromatin domains are formed in space and time. Positive feedback, where histone-modifying enzymes are attracted to chromosomal regions displaying the modification they catalyse, is believed to drive the formation of these domains; however, few quantitative studies are available to assess this hypothesis. Here we quantified the *de novo* establishment of a naturally occurring ∼20-kb heterochromatin domain in fission yeast through single-cell analyses, measuring the kinetics of heterochromatin nucleation in a region targeted by RNAi and its subsequent expansion. We found that nucleation of heterochromatin is stochastic and can take from one to ten cell generations. Further silencing of the full region takes another one to ten generations. Quantitative modelling of the observed kinetics emphasizes the importance of local feedback, where a nucleosome-bound enzyme modifies adjacent nucleosomes, combined with a feedback where recruited enzymes can act at a distance.

## Abstract (web summary)

Chromosomes contain large heterochromatin domains. Here, the authors measure the kinetics of heterochromatin formation in fission yeast and show both global and local feedbacks by nucleosome-bound enzymes are important for formation and stability of the large heterochromatin domains.

## Graphical abstract

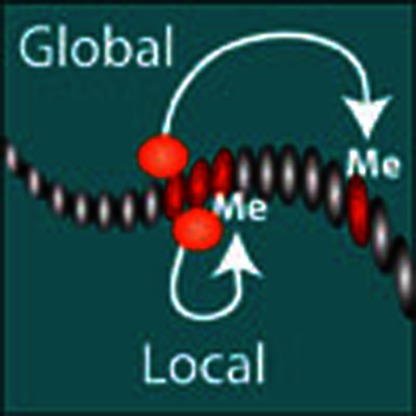

Many histone-modifying enzymes bind their modified substrates. This binding, together with other types of positive feedback that generally accompany it, is believed to facilitate the spreading of nucleosome modifications along chromosomes from nucleation sites and the modification of newly deposited nucleosomes following DNA replication, perhaps participating in epigenetic memory. In these models, an enzyme attracted to a modified nucleosome modifies nucleosomes in the vicinity. In contrast to the molecular interactions that lead to the deposition of histone modifications, kinetic aspects of chromatin formation that would allow testing such models have seldom been reported.

A well-documented example of positive feedback is in heterochromatin formation, where methyltransferases of the KMT1 family bind methylated histone H3K9 (H3K9me) with chromodomains and methylate H3K9 with their SET domains^1^,^2^,^3^, establishing a major type of repressive heterochromatin whose dynamics are critical to development, cell differentiation and the maintenance of genome integrity^4^,^5^,^6^,^7^,^8^,^9^,^10^,^11^,^12^,^13^. In the fission yeast *Schizosaccharomyces pombe*, mutations in the chromodomain of the KMT1 Clr4 that reduce the affinity of Clr4 for H3K9me alleviate H3K9me *in vivo*, underscoring the importance of Clr4 self-recruitment for heterochromatin formation^2^,^3^,^14^,^15^. There, self-recruitment is fostered by several histone deacetylases (HDACs), RNA interference (RNAi) factors and an H3K4 demethylase (Lid2) that together indirectly promote H3K9 methylation by Clr4 (refs 3, 15, 16, 17, 18, 19, 20, 21, 22). Likewise, antagonistic histone-modifying enzymes benefit from positive feedback. Histone acetyltransferases, which antagonize HDACs, bind to acetylated nucleosomes via bromodomains, to promote expressed acetylated chromatin states^23^. Demethylases that remove methyl groups from H3K9me bind to nucleosomes bearing active state modifications via double Tudor or PHD domains, or participate in histone-binding protein complexes. This is the case for JMJD2A^24^,^25^, PHF8 (ref. 26), ceKDM7A^27^ and PHF2 (ref. 28) in higher eukaryotes, and for *S. pombe* Lsd1 (refs 29, 30, 31).

Chromatin landscapes are dynamically shaped by such opposing activities at all times, with widespread changes in histone modification profiles underlying changes in gene expression programmes^4^,^5^,^6^,^7^,^8^,^9^,^10^,^11^,^12^,^13^. Not surprisingly, temporal transition states as occur during cell differentiation, early embryogenesis or at the onset of disease have been more difficult to characterize experimentally than terminally differentiated chromatin, because some changes affect only small numbers of cells or do not occur synchronously in populations. Here, to gain insights into the kinetics of chromatin formation, we applied single-cell measurements to capture the non-equilibrium states of a naturally occurring H3K9me domain of *S. pombe* as the domain transitions from euchromatin to heterochromatin.

In *S. pombe*, all detectable H3K9me depends on Clr4 (ref. 2, 32). Clr4 is found in the ClrC complex, which catalyses the formation of large heterochromatic domains at centromeres, telomeres and in the mating-type region (Zhang *et al*.^3^ and references therein). The system has been extensively studied. Among its many advantages is the fact that fission yeast lacks DNA methylation on cytosines, eliminating a common caveat in investigations of the role of histone modifications in epigenetic inheritance.

In the region used for our measurements, the mating-type region, heterochromatin occupies ∼20 kb between the two boundary elements *IR-L* and *IR-R*^33^,^34^ (Fig. 1), silencing the *mat2-P* and *mat3-M* mating-type loci and the entire intervening region^34^,^35^,^36^,^37^. RNAi is believed to nucleate heterochromatin formation through an ∼4-kb element located between *mat2-P* and *mat3-M*, *cenH*. RNAi contributes strongly to heterochromatin formation at *S. pombe* centromeres^38^,^39^ where many repeats homologous to *cenH* are found^40^ and, to a lesser extent, at telomeres where a few repeats are found^41^. Non-coding RNA transcribed from the repeats are amplified by an RNA-dependent RNA polymerase, cleaved by Dicer into small interfering RNAs, and incorporated into an Argonaute family protein, Ago1, in the RITS complex. Multiple physical interactions take place between RNAi complexes and ClrC, leading to the recruitment of ClrC to regions where RNAi is active and, conversely, to the recruitment of RNAi to regions with H3K9me, in self-inforcing loops^3^,^20^,^21^,^22^,^42^,^43^.

Deletion of RNAi factors do not result in immediately discernable heterochromatin loss in the mating-type region, unlike for centromeres^38^,^44^,^45^, but the *de novo* heterochromatin formation in the mating-type region is slow in RNAi mutants^44^,^45^,^46^,^47^ and *cenH* mediates heterochromatin formation at ectopic locations in an RNAi-dependent manner^44^,^48^, indicating RNAi acting at *cenH* catalyses the establishment of heterochromatin. In addition to RNAi, the Atf1/Pcr1 dimer binding to a site between *cenH* and *mat3-M* also contributes to heterochromatin formation^49^. Mutants lacking *cenH*, the *ΔK* mutants, adopt a bistable phenotype alternating between two epigenetic states, one state with the same defects as *clr4Δ* mutants and the other state like wild type^50^,^51^. Switches between the two states occur at rates of ∼1/2,000 cell divisions^51^. Together with the phenotypes of RNAi mutants and with the many ties established between RNAi and *cenH* repeats elsewhere in the genome, the bistability of *ΔK* strains suggests that deletion of *cenH* reduces the rate of heterochromatin establishment by removing an RNAi-dependent element that normally facilitates Clr4 recruitment. RNAi would increase the rate of establishment from ∼1/2,000 cell division in cells lacking *cenH* to some higher rate that we set up to measure.

The *in vivo* kinetic measurements presented here were conducted with the goal of quantitative modelling. The notion that modified nucleosomes promote inheritance and spreading of modifications by attracting modifying enzymes is common in the literature but tests of these models have been hampered by a lack of quantitative data. Our measurements allow us to test conditions for which positive feedback in the recruitment of histone-modifying enzymes permits heterochromatin formation and inheritance, expanding on our previous modelling^52^,^53^,^54^,^55^ to uncover basic principles underlying eukaryotic chromatin formation. Specifically, our new data and modelling show how combined local reactions, where an enzyme recruited to a nucleosome modifies the neighbour nucleosomes, and global reactions, where some recruited enzymes modify more distant nucleosomes, contribute to the establishment of large heterochromatic domains. The reactions result in long-lasting heterogeneity in cell populations that are in the process of changing epigenetic state.

## Results

### Heterochromatin is established slowly in populations

**Figure 1:**
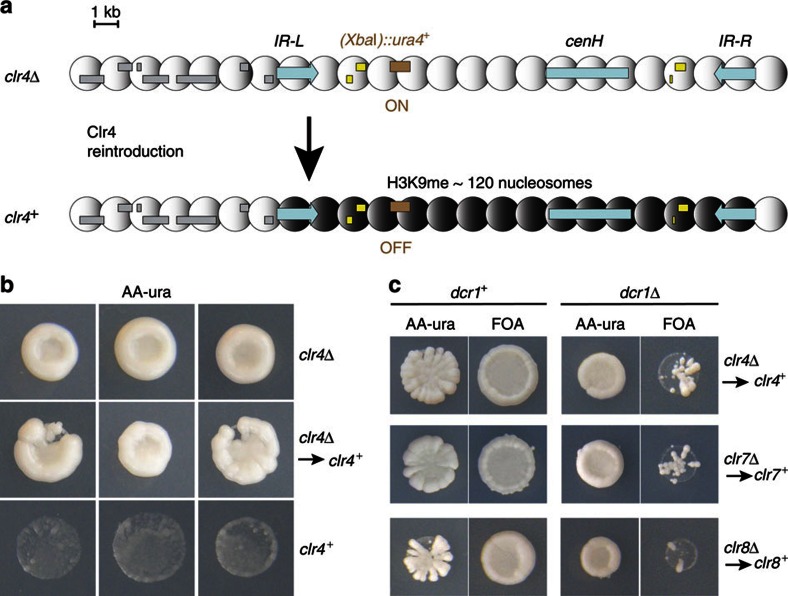
Slow establishment of heterochromatic silencing by the H3K9 methyltransferase Clr4. (**a**) Mating-type region. Nucleosomes between the *IR-L* and *IR-R* boundaries are methylated by Clr4, silencing gene expression. The chromosomal location of the *(XbaI)::ura4*^+^ reporter is shown. *cenH*: ∼4.2 kb region with centromere homology; grey boxes: ORFs; yellow boxes: silent mating-type information at *mat2-P* and *mat3-M*. (**b**) *de novo* silencing of *(XbaI)::ura4*^+^ by Clr4. Colonies formed by spores on rich medium were replicated onto medium lacking uracil. All colonies shown contain the *(XbaI)::ura4*^+^ reporter. A phenotypic gap is observed following re-introduction of *clr4*^+^into *clr4Δ* cells (middle panels) allowing *clr4*^+^cells to grow in the absence of uracil. The three colonies shown on the last row (*clr4*^+^) are genetically identical to the colonies above them (*clr4Δ to clr4*^+^) but their heterochromatin is fully established. (**c**) Dicer facilitates the establishment of heterochromatic silencing by Clr4, Clr7 and Clr8. Wild-type alleles were re-introduced into deletion strains by genetic crosses as indicated. Silencing of *(XbaI)::ura4*^+^ permits growth on 5-fluoroorotic acid (FOA). Establishement of *(XbaI)::ura4*^+^ silencing is more efficient in the *dcr1*^+^ than in the *dcr1Δ* progeny of each cross.

Reporter genes inserted between the *IR-L* and *IR-R* boundaries are silenced by heterochromatin as shown in Fig. 1 for an insertion of *ura4*^+^ near *mat2-P*, *(XbaI)::ura4*^+^ (ref. 37). *(XbaI)::ura4*^+^ cells do not grow in the absence of uracil and are resistant to 5-fluoroorotic acid, a toxigenic substrate of the orotidine 5′-phosphate decarboxylase encoded by *ura4*^+^. This silencing is alleviated in mutant cells lacking Clr4 (ref. 37). We monitored the establishment of Clr4-mediated silencing by re-introducing a functional *clr4*^+^ gene into *(XbaI)::ura4*^+^ *clr4Δ* cells through a genetic cross. *(XbaI)::ura4*^+^ *clr4Δ* cells were crossed with *mat1-PΔ17::LEU2 clr4*^+^ cells whose mating-type region is easy to follow in crosses. Spores were micromanipulated into a grid on rich medium, allowed to form colonies and colonies were replicated onto selective media to assign genotypes and to assay *(XbaI)::ura4*^+^ expression. As expected, *(XbaI)::ura4*^+^ remained derepressed in the *(XbaI)::ura4*^+^ *clr4Δ* progeny of the cross (Fig. 1b; first row). Surprisingly, *(XbaI)::ura4*^+^ also remained derepressed in the *(XbaI)::ura4*^+^ *clr4*^+^ progeny of the cross (Fig. 1b; second row). In a control cross where a *(XbaI)::ura4*^+^ *clr4*^+^ strain with fully established heterochromatin was used instead of the *(XbaI)::ura4*^+^ *clr4Δ* strain, *(XbaI)::ura4*^+^ remained repressed in all progeny, indicating meiosis does not alleviate repression (Fig. 1b; third row). In the *clr4*^+^ re-introduction experiment, some cells still displayed a mutant Ura^+^ phenotype >20 generations after they became genetically wild type (Fig. 1b; second row). This long phenotypic gap indicates that H3K9me is not established efficiently, certainly not at each cell division, in a naive mating-type region lacking all H3K9me.

We compared establishment of silencing of *(XbaI)::ura4*^+^ following re-introduction of *clr4*^+^ in *dcr1*^+^ and *dcr1Δ* cells. A similar experiment was conducted previously in *dcr1Δ* cells, where slow establishment was observed; however, the experiment did not examine a wild-type *dcr1*^+^ background for comparison, rendering it difficult to judge the extent to which lack of RNAi slows down establishment^44^. Here, genetic crosses were conducted in two ways that produced identical results. First, a *clr4*^+^ re-introduction experiment was performed in a *dcr1Δ* background, where both parents were *dcr1Δ*, and compared with a parallel cross in which both parents were *dcr1*^+^. Second, *(XbaI)::ura4*^+^ *clr4Δ* cells were crossed with *clr4*^+^ *dcr1Δ* cells and the *(XbaI)::ura4*^+^ *clr4*^+^ *dcr1Δ* progeny, lacking RNAi, were compared with the *(XbaI)::ura4*^+^ *clr4*^+^ *dcr1*^+^ progeny, proficient for RNAi. Delayed establishment of 5-fluoroorotic acid resistance and uracil auxotrophy in the *dcr1Δ* progeny (Fig. 1c) showed that heterochromatic silencing following re-introduction of Clr4 is slowed down by the absence of RNAi. Further, two subunits of the ClrC complex, respectively, Clr7 (a.k.a. Raf2 or Dos2) and Clr8 (a.k.a. Raf1 or Dos1), behaved as Clr4 in re-introduction experiments in both *dcr1*^+^ and *dcr1Δ* backgrounds (Fig. 1c). In addition, the wild-type levels of mating-type switching and sporulation were also acquired with a delay in *clr4*^+^ re-introduction experiments, consistent with slow heterochromatin establishment in both RNAi-proficient and -deficient cells, with a more pronounced delay in RNAi mutants (Supplementary Fig. 1). These results confirm that RNAi normally facilitates the establishment of H3K9me in the mating-type region by the ClrC complex as already shown by others^45^,^46^. Importantly, these results also show that the RNAi-mediated establishment of heterochromatin in wild-type cells is very inefficient. Slow establishment in a wild-type background has, to our knowledge, not been reported previously, because previous studies examined establishment at later time points following the re-introduction of *clr4*^+^.

### Stochastic establishment and clonal inheritance

**Figure 2:**
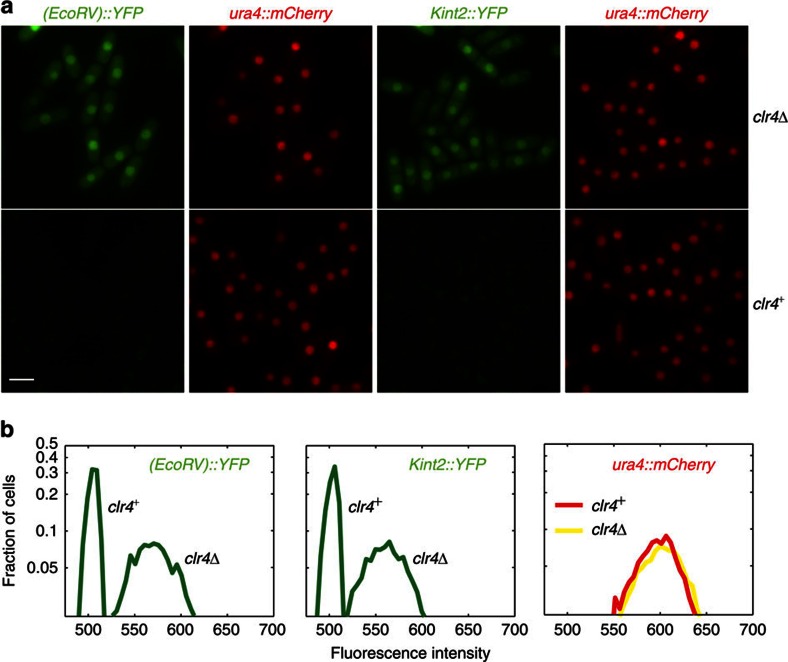
Development of fluorescent reporters for single-cell measurements. A nuclear-targeted YFP under control of the *ura4*^+^ promoter was expressed from two sites in the mating-type region, respectively, *(EcoRV)* near the centromere-distal edge of the heterochromatic region and *kint2* within the *cenH* element. The location of these sites is depicted in Figs 3 and 4. mCherry was expressed in the same cells from the *ura4*^+^ euchromatic locus. (**a**) Fluorescence of *kint2::YFP*, *(EcoRV)::YFP* and *ura4::mCherry* in *clr4Δ* and *clr4*^+^ cells. Scale bar, 5 μm. (**b**) Distribution of fluorescence intensities for the same strains, obtained by measuring nuclear fluorescence in microscopy images.

**Figure 3:**
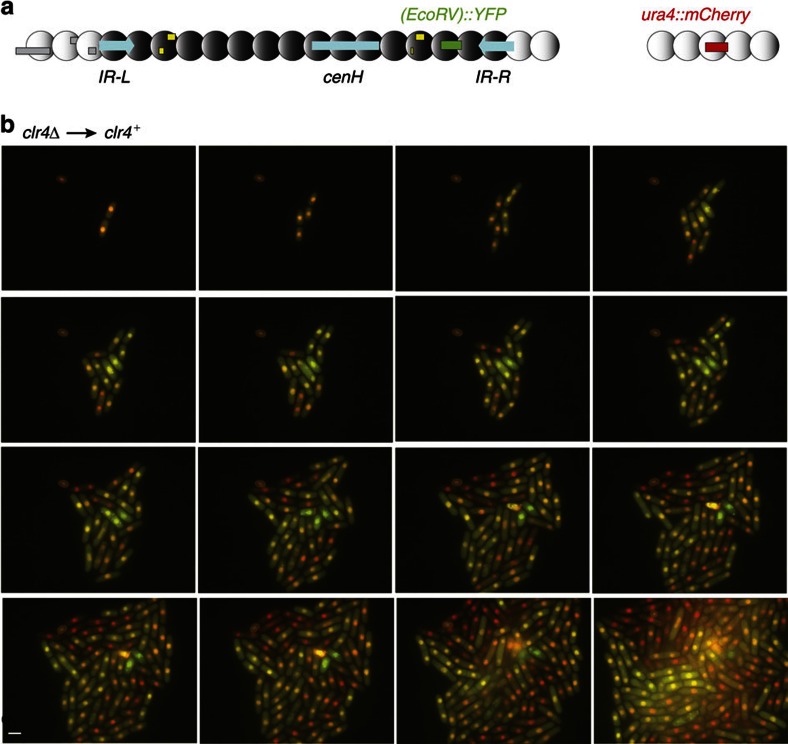
Fission yeast cells dividing under a fluorescence microscope establish gene silencing. (**a**) Fluorescent reporters used for imaging. (**b**) Overlay of the mCherry and YFP channels, starting at the first cell division following the re-introduction of *clr4*^+^ through a cross. At first, cells express both YFP and mCherry (yellow-green signal). As they divide, a few cells silence YFP (red signal). The silenced state is inherited clonally in their progeny. Scale bar, 5 μm. Additional timepoints and separate channels are shown in Supplementary Movies 1-3.

To assess the behaviour of individuals in populations of cells undergoing heterochromatin establishment, we inserted a gene encoding the yellow fluorescent protein (YFP) near *mat3-M* (*(EcoRV)::YFP*; Figs 2 and 3a). As a reference, we inserted a gene for the fluorescent protein mCherry in euchromatin, at the *ura4* locus (*ura4::mCherry*). Expression of both YFP and mCherry was controlled by the *ura4* promoter and terminator of transcription, and both proteins were targeted to the nucleus by an SV40 nuclear localization sequence. This experimental setup is similar to the setup used in *Saccharomyces cerevisiae* by Xu *et al*.^56^ As was the case for the *(XbaI)::ura4*^+^ reporter used in Fig. 1, *(EcoRV)::YFP* was repressed by heterochromatin in wild-type cells but it was expressed in a *clr4Δ* mutant (Fig. 2). In *clr4Δ* cells, a unimodal fluorescence distribution was observed for YFP, the distribution did not overlap with the fluorescence distribution in *clr4*^+^ cells. The expression of *ura4::mCherry* did not differ between *clr4*^+^ and *clr4Δ* cells (Fig. 2).

**Figure 4:**
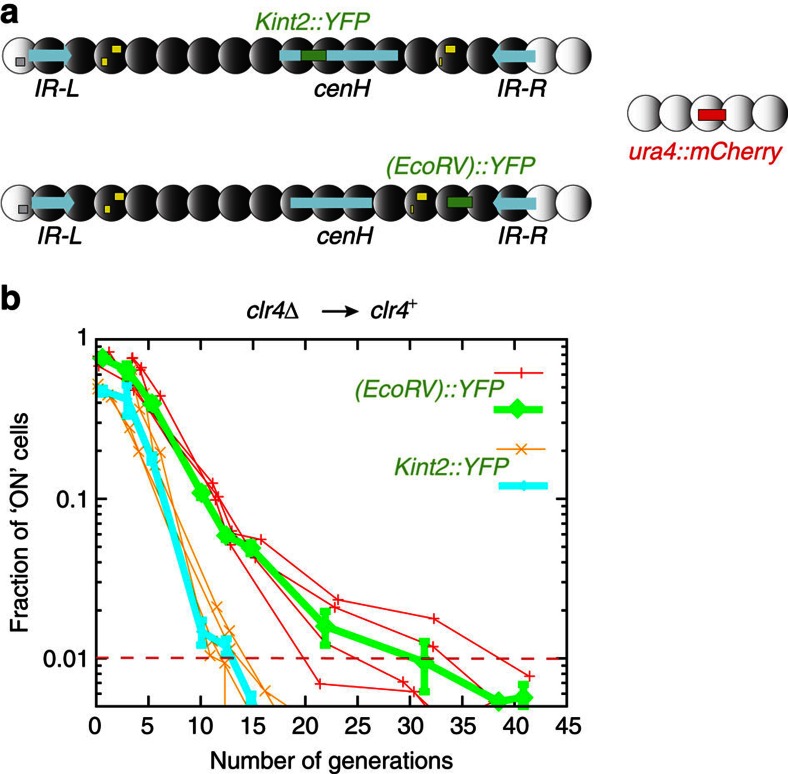
The kinetics of heterochromatin establishment are faster within *cenH* than outside *cenH*. (**a**) Location of the *kint2::YFP* and *(EcoRV)::YFP* reporters used in the experiment. These reporters are also shown in Fig. 2. (**b**) Establishment of heterochromatic silencing at *kint2::YFP* and *(EcoRV)::YFP* in re-introduction experiments, starting at the first division following re-introduction of *clr4*^+^. Fluorescence intensity was measured in microcopy images sampling liquid cultures at the indicated time point. The experiment was conducted in quadruplicate (thin lines) and the data were binned (thick lines). Error bars represent the s.d. of the values in each bin.

As for *(XbaI)::ura4*^+^, the expression of *(EcoRV)::YFP* was assayed following re-introduction of the wild-type *clr4*^+^ gene through a genetic cross but, unlike for *(XbaI)::ura4*^+^, cells could be interrogated starting at their first division following the re-introduction of *clr4*^+^. Prototrophic markers were used to select the desired progeny. *arg12*^+^ was used to select *clr4*^+^ and *ura4*^+^ integrated at the *Xmn*I site on the euchromatic side of *IR-L*^51^ was used to select the mating-type region with *(EcoRV)::YFP*. *(EcoRV)::YFP clr4*^+^ spores were induced to germinate by placing the spores at 33 °C in minimal medium. Germination occurs in a synchronous manner under these conditions. It produces cells that can be examined right away in a temperature-controlled growth chamber under a fluorescence microscope (Fig. 3), or allowed to proliferate in liquid cultures with frequent sampling (Fig. 4). Establishment of H3K9me in the mating-type region resulted in silencing of YFP. Time-lapse series were obtained in which germinated spores divided to form microcolonies, examples of which are shown in Fig. 3b and Supplementary Movies 1–3. These series indicated that silencing of YFP occurred stochastically at a rate of ∼1 in 8 cell divisions (for example, 9 events in 72 divisions of ‘ON' cells in Supplementary Movies). Once established, the silenced state was transmitted to the mitotic progeny. Crowding imposed a limit to the number of generations for which cells could be imaged in the growth chamber. Liquid cultures were used instead, to examine cells for longer periods following spore germination (Fig. 4). Growth curves were established and the proportion of cells expressing YFP was determined every few hours by fluorescence microscopy, confirming the estimated rate of *(EcoRV)::YFP* silencing. Although some YFP protein is likely to be transmitted from one generation to the next, the protein is diluted exponentially as cells keep dividing. Time-lapse microscopy, where it is possible to trace back turn-off points to single cells in pedigrees, indicates that this transmission has little effect on the estimated rate of silencing. Using a homologous reporter system in *S. cerevisiae*, Xu *et al*.^56^ showed that similar measurements were independent of reporter stability.

The silencing kinetics of *(EcoRV)::YFP* indicate that the establishment of heterochromatin results from rare stochastic events rather than from slow changes building up gradually over many generations in a synchronous manner for all cells in the population. A slow and steady increase would lead to a large frequency of concomitant switches, which were not observed. Although low, the rate of establishment at *(EcoRV)* in wild type is ∼150-fold higher than in the *ΔK* mutant^51^.

### Establishment within the RNAi-dependent *cenH* element

We investigated whether the establishment of heterochromatin at the proposed target for RNAi, *cenH*, differs from neighbouring region with a strain where YFP was expressed from *cenH* (*Kint2::YFP*) instead of *(EcoRV)::YFP* (Figs 2 and 4). In *clr4Δ* cells, YFP expression was similar at *Kint2* and (*EcoRV*) (Fig. 2). Following re-introduction of Clr4, silencing at *Kint2::YFP* occurred faster than silencing at *(EcoRV)::YFP* measured in a parallel experiment (Fig. 4), indicating that heterochromatin might be first established within *cenH*, and that the modifications would subsequently spread to the rest of the region.

### Heterochromatin spreads outwards from *cenH*

**Figure 5:**
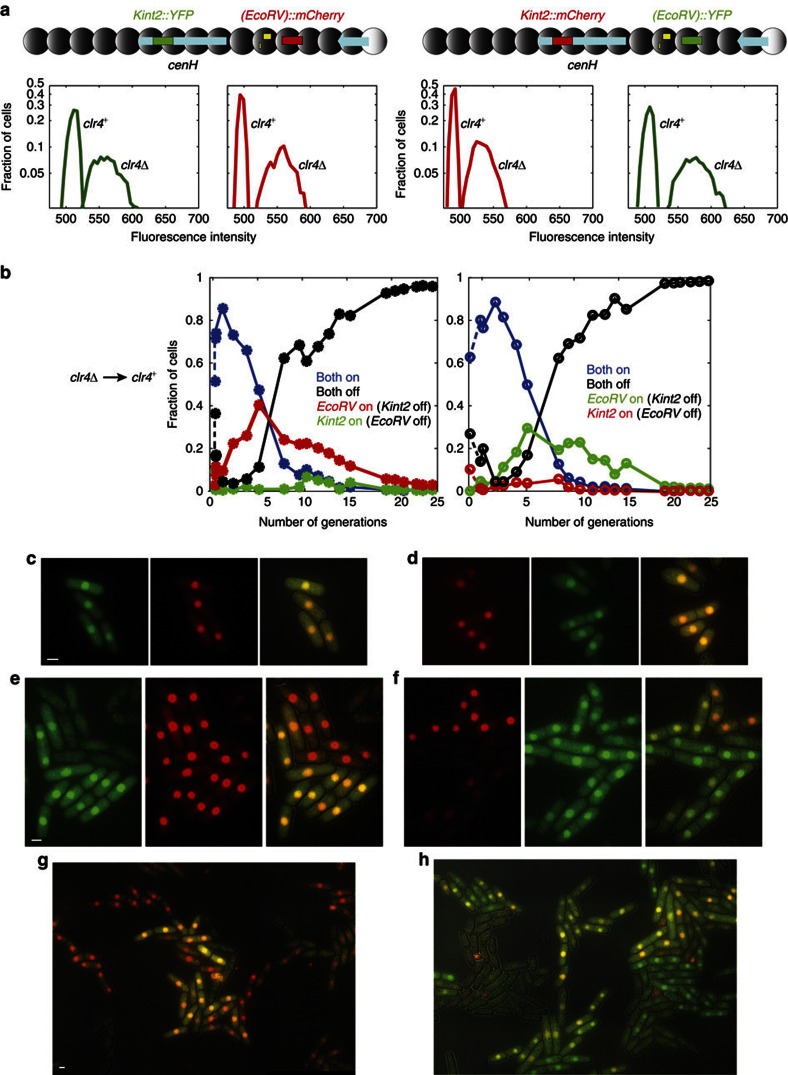
Spreading of heterochromatin outward from *cenH*. (**a**) Fluorescent reporters used in the experiment. Cells with *Kint2::YFP (EcoRV)::mCherry* are shown on the left in all panels; *Kint2::mCherry (EcoRV)::YFP* cells are shown on the right. (**b**) YFP and mCherry fluorescence was measured by microscopy at the indicated time points following the re-introduction of *clr4*^+^ examining >1,000 cells for each data point. Some uncertainty for the initial timepoint, due to the difficulty in counting germinating spores, is indicated by dashed lines. Histograms for the fluorescence distributions are shown in Supplementary Fig. 2. (**c**-**h**) Representative images. (**c**,**d**) At the second division following spore germination, the vast majority of *clr4*^+^ cells express both reporters. (**e**,**f**) As cells divide (generation 5), silencing of the reporter at *Kint2* (whether *Kint2::YFP* on the left or *Kint2::mCherry* on the right) precedes silencing of the reporter at *(EcoRV)*. (**d**,**f**) Generation 7. Scale bars, 2 μm.

Whether *Kint2* and *(EcoRV)* are silenced consecutively was investigated with strains with two linked fluorescent reporters. One strain contained the *mCherry* gene at the *Kint2* site within *cenH* and the *YFP* gene at *(EcoRV)* near *mat3-M* (Fig. 5a). The other strain had the reversed order of markers. Both *YFP* and *mCherr*y were controlled by the *ura4*^+^ promoter and terminator of transcription, and the proteins were targeted to the nucleus. In *clr4Δ* cells, YFP and mCherry were expressed from both locations and the YFP (respectively mCherry) signal was of similar intensity whether expressed from *Kint2* or from *(EcoRV)* (Fig. 5a). *clr4*^+^ was re-introduced as above through a cross where only the *clr4*^+^ progeny with the labelled mating-type region were allowed to germinate and the expression of YFP and mCherry was monitored over time by fluorescence microscopy, sampling liquid cultures (Fig. 5b–h). Silencing occurred with the same kinetics as observed previously (Fig. 4 and Supplementary Movies 1–3). The rates of silencing were determined by fitting an exponential function (Matlab) to the number of ‘ON' cells as a function of time. The ±values correspond to the 95% confidence interval resulting from fitting. The rates of silencing at *Kint2* were 0.20±0.04 silencing event per cell division for YFP and 0.25±0.02 for mCherry. The rates of silencing at *(EcoRV)* were 0.12±0.02 measured with mCherry or 0.14±0.02 for YFP. Silencing at *(EcoRV)* occurred with a delay compared with *Kint2* and, with rare exceptions, only in cells that had also silenced the reporter at *Kint2* (Fig. 5b). Five generations after re-introduction of *clr4*^+^, for example, ∼50% of cells expressed both reporters, ∼15% had silenced both reporters, ∼35% had silenced *Kint2* but not *(EcoRV)* and <1% had silenced *(EcoRV)* but not *Kint2*. The same kinetics were observed for the *Kint2::YFP (EcoRV)::mCherry* strain (where YFP was silenced before mCherry) and for the *Kint2::mCherry (EcoRV)::YFP* strain (where mCherry was silenced before YFP), ruling out that different fluorescent properties or protein half-lives might cause the differences observed for the two locations (Fig. 5). At each time point the probability of silencing *(EcoRV)* was much higher (in the order of 100-fold higher) in cells that had silenced *Kint2* than in cells that had not silenced *Kint2*, indicating that the two events are not independent, and that heterochromatin spreads from *Kint2* to *(EcoRV)*. Together, these observations show that the establishment of heterochromatic silencing occurs first at *cenH*, and that nucleation of heterochromatin at *cenH* facilitates heterochromatin formation in the rest of the mating-type region.

### Quantitative modelling of heterochromatin formation

**Figure 6:**
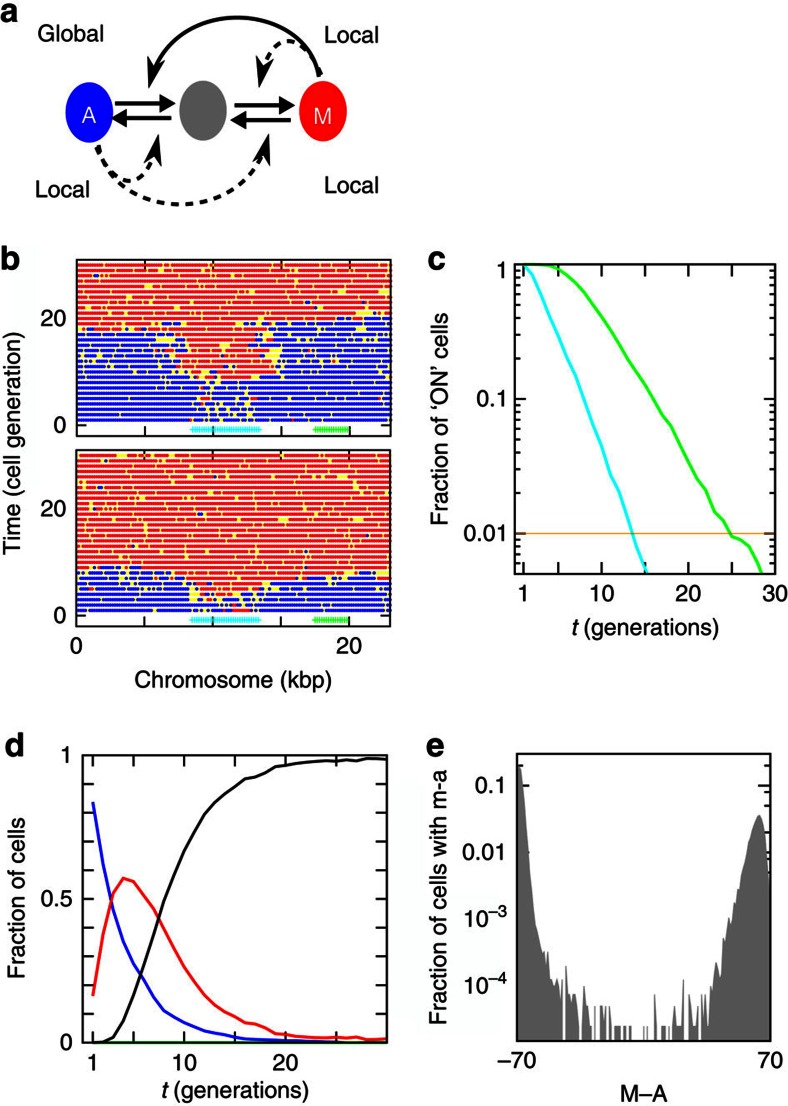
Combinations of global and local nucleosomal cross-talks recapitulate the observed kinetics. (**a**) Positive feedbacks in the recruitment of histone-modifying enzymes. Nucleosomes exist in three states, methylated (M; red), unmodified (U; grey), or acetylated (A; blue). Methylated and acetylated nucleosomes recruit modifying enzymes capable of modifying the immediate neighbours of the nucleosome to which they bind (thin dashed arrows: local feedback) and, for A to U, more distant nucleosomes as well (thick arrow: global feedback). The evolution of a 23-kb region representing the mating-type region with two reporters was simulated, using parameters and a source of methylation at *cenH* (cyan) as described in the text. (**b**) Two model simulations with identical parameters of the dynamics in nucleosome modification profile over the 23-kb mating-type region following the re-introduction of Clr4 at time 0. (**c**) Times of silencing for the *Kint2* (cyan) and (*EcoRV)* (green) positions in the simulations occurred with the kinetics measured *in vivo* at these two locations. (**d**) Fraction of cells with none, one or both genes active as function of time. (**e**) Distribution of epigenetic states for the *ΔK* mutant with an epigenetic stability compatible with experimental measurements.

We previously modelled how modified nucleosomes can participate in epigenetic memory by attracting read–write enzymes that catalyse the modifications they display^52^,^53^,^54^. Here we used core elements from Dodd *et al*.^52^ to model heterochromatin establishment in wild-type cells, introducing the notion that RNAi facilitates Clr4 recruitment within *cenH* (Fig. 6). In this modified model, the mating-type region is represented by 140 nucleosomes arranged in a line. As in Dodd *et al*.^52^ nucleosomes exist in three states: unmodified (U), heterochromatic (methylated at H3K9; M) or euchromatic (acetylated; A). Nucleosomes are exposed to modifying enzymes that may be recruited or be spontaneously acting as shown in Fig. 6a. Changes in chromatin structure result from (1) iterating the ‘nucleosome modification procedure' described below and (2) periodically mimicking cell division by replacing half the nucleosomes in the region with unmodified nucleosomes.

In the nucleosome modification procedure^52^, a randomly picked ‘modifying' nucleosome is allowed to interact with a ‘substrate' nucleosome. No change is made if the two nucleosomes are in the same state, otherwise the substrate nucleosome is moved one step towards the state of the modifying nucleosome with a rate reflecting the ability of nucleosomes to directly or indirectly recruit histone-modifying enzymes. In addition, nucleosomes may be exposed to modifying enzymes that act independently of the other nucleosomes within the considered system. All reactions are assigned rate parameters.

The system is iterated in discrete events using a Gillespie algorithm with the assigned rate parameters. Each selected event defines one attempted modification process. In case the event is a spontaneous conversion, for example, of A to U, one selects one nucleosome randomly and makes the conversion if the nucleosome is in the appropriate state (in this case an A state). In case the chosen reaction is a read–write event (for example, M-state-catalysed conversion of A to U), one randomly selects a potential modifying nucleosome and a target nucleosome. If this pair of nucleosomes is not in states that permit the chosen recruitment (for example, if the recruiting nucleosome is not in M), no change is made to the system. If both recruiting and target nucleosomes match the chosen reaction, the target nucleosome is modified.

Two modes of recruitment interactions are permitted, local or global^53^. For local feedback, an enzyme recruited by a modified nucleosome can modify only the immediate neighbours of that nucleosome. Hence, only nucleosomes that are adjacent to the modifying nucleosome can be picked to function as substrates. For global feedback, the enzyme recruited by a modified nucleosome can modify any nucleosome in the system with a probability inversely proportional to the distance between the two nucleosomes. In this case, any nucleosome in the system can be picked as a potential substrate, according to the chosen distance probability rule.

Noticeably, the attempt rate is not the same as the success rate, because a reaction is only successful when the chosen pair of nucleosomes are in states that match the assigned process. For example, when the number of A or M is small, the actual number of reactions where M facilitates a change from A to U is small. Therefore, this particular reaction will mostly occur when the system is in the transition between an A-dominated state and an M-dominated state. In the Supplementary Notes 1–3 we describe model variants and simulations in detail.

In the version of the model shown in Fig. 6 only M-state-catalysed conversion of A to U was considered global, whereas the three other processes were local. Each process was attempted at a rate of 100 times per nucleosome between cell divisions, except the M-state-catalysed U to M conversion that was attempted 30 times per nucleosome. In addition to these recruited reactions, we allowed small rates of spontaneous changes (2.5 per nucleosome per generation for all conversions) that represent chance encounters with histone-modifying enzymes external to the system.

To reflect the ability of *cenH* to facilitate H3K9me, a source of modification was introduced at *cenH*, consisting of 29 nucleosomes for which spontaneous conversions of A to U were attempted at a rate of 16 attempts per nucleosome per cell generation. This source together with the recruitment rules described above recapitulated the kinetics of heterochromatin establishment within and outside *cenH* (Fig. 6b–d). Following removal of the source, the model correctly reproduced the bistability of the remaining system of 70 nucleosomes (Fig. 6e), that is, the model also recapitulates the phenotypes of the *ΔK* mutants.

The above model is one example of a wider class of recruitment scenarios for nucleosome-mediated epigenetics. Supplementary Note 3 explores variant models where not all modification reactions are catalysed by read–write enzymes, models with other combinations of local and global recruitments, and also models with more than three nucleosome states. Apart from the need for long-range recruitment reactions and cooperativity formulated by Dodd *et al*.^52^, the combination of model testing and our new timing experiments teaches us the following: (i) Recruitments on all modifications greatly enhance the robustness of epigenetic bistability against spontaneous conversions. (ii) Most recruitment reactions should be acting only locally, to reproduce the sequential silencing of the two reporters. (iii) Within three-state models, one needs long-range recruitment on the A to U modification acting from the silenced M state, to obtain the observed combination of robust bistability (to spontaneous conversions) and differential timing (between *cenH* and the whole system). (iv) The four-state model provides a double-switch interpretation for the two consecutive silencing events.

The model variation with additional modification steps deserves special attention. It is justified by the fact that each nucleosome contains two copies of each histone, that each lysine can be multiply methylated and also that the binding of proteins to methylated nucleosomes may be cooperative, as proposed in the case of Swi6 (ref. 57). Further, the various methylation states of H3K9 *in vivo* have different affinities for chromodomain proteins^3^,^58^. We find that a fourth step provides increased robustness to non-recruited modifications (see Supplementary Note 4). A second and remarkable outcome of the four-state model is an alternative interpretation of the spreading of silencing marks where spreading occurs intermittently from *cenH* to the remaining system. Silencing of *cenH* represents one stochastic switch, followed by a second switch that provides substantial cell-to-cell heterogeneity in the time needed to silence the whole region. The experimental hallmark of such a second switch is an exponential distribution of the time difference between the first and the second silencing event.

## Discussion

Although the molecular interaction networks that permit the association of histone-modifying enzymes to chromatin are often well known, kinetic parameters are not. Here we determined the kinetics of *de novo* establishment of heterochromatin in the mating-type region of fission yeast. We found that establishment occurs stochastically in populations. Stochastic establishment and epigenetic inheritance result in heterogeneous populations where distinct epigenetic states coexist for ∼20 generations. Mathematical modelling, used to test conditions for which positive feedback in the recruitment of histone-modifying enzymes to chromatin recapitulates the observed rates of heterochromatin nucleation and spreading, provides new insights into the mechanisms of heterochromatin formation.

A first point that modelling helps understand is the large cell-to-cell variation in the time of heterochromatin establishment. Conceivably, the establishment of a chromatin state in a large chromosomal domain results from initiating events followed by spreading that lead to a stable conversion of the entire region. The observed stochasticity of establishment might reflect a switch-like silencing of *cenH* followed by a stochastic propagation. Our model shows how propagation relying on the recruitment of histone-modifying enzymes by modified nucleosomes would result in stochastic establishment with a characteristic timescale of a few cell generations. Heterogeneity in gene expression and chromatin structure is observed in populations of differentiating cells (reviewed by, for example, Tee and Reinberg^59^). Our observations indicate how heterogeneity resulting from cells engaging into diverse differentiation programmes might be compounded by temporal stochasticity in the establishment of chromatin structures. The stochastic establishment of H3K9me chromatin domains might in itself be a key determinant of some differentiation programmes, as it has been proposed to underlie important cell-fate decisions in mammals (reviewed in Magklara and Lomvardas^13^). Stochasticity in the establishment of chromatin structures might generally contribute to developmental variation and diversity in adult populations and it might account for the incomplete penetrance of some genetic defects where only a fraction of the population is affected by a mutation resulting in an otherwise severe phenotype (for example, Morgan *et al*.^60^). Cell-to-cell heterogeneity in the nucleation of H3K27me3 at the *Arabidopsis* FLC locus ensures a quantitative response to cold exposure at the population level^61^,^62^, which, as for the present system, is epigenetically stored in *cis* in the chromosomes of individual cells^50^,^51^,^63^.

Mechanistically, variations on our model highlight the complementary effects of global and local nucleosomal recruitments in chromatin formation. Locally acting recruitments reflect steric constraints for reactions in which a nucleosome-bound enzyme might not be able to catalyse the modification of a distant nucleosome, while globally acting recruitments account for reactions that might not be similarly constrained. Here we found that combinations of global and local effects were necessary to model the observed combination of nucleation rate and subsequent kinetics of spreading. Feedback interactions limited to local contacts, as proposed for the modelling of a smaller artificial system^64^ could not account for the observed exponential distribution of turn-off times at the *cenH* region. The ∼5–15 h it subsequently takes to spread along the 25 nucleosomes between *cenH* to EcoRV is about a factor ten faster than the 0.18 nucleosomes per hour observed by Hathaway *et al*.^64^ in a mammalian system with its much longer cell generation. Thus, in units of cell generation the timescale for the spreading part of the dynamics might be comparable between these different organisms. However, the large cell-to-cell variation in the time of spreading is compatible with an alternative scenario of intermittent dynamics that has a characteristic timescale of about five generations. The possibility for this scenario is in particular supported by the four-state variant of our model (Supplementary Note 4).

It has been pointed out before that purely global feedback interactions would not permit the confinement of chromatin states to specific chromosomal domains^53^. On the other hand, strictly local interactions fail to produce bistable systems^52^,^53^ and they do not lead to the characteristic plateau shape of large heterochromatin domains^64^. In contrast, combinations of global and local recruitment allow the formation of bounded domains^53^ and account for the diverse kinetics of heterochromatin nucleation, spreading and inheritance we observed, including the behaviour of bistable mutants (Fig. 6).

Molecular mechanisms for both local and global feedback have been documented experimentally. In the case of Clr4 and for the mouse methyltransferases GLP and G9a, binding to trimethylated H3K9 stimulates the methylation of nearby nucleosomes *in vitro*^65^,^66^. Structural studies have also shown the importance of steric effects for proteins that are both readers and writers of histone modifications^26^,^27^. In the system we modelled, global positive feedback might be provided by RNAi spreading heterochromatin through transcription and associations with RNA^49^,^67^, through the polymerization of Tas3, a RITS subunit^68^ or by the association of ClrC with DNA polymerases^69^. As the association of HDACs with H3K9me domains can occur indirectly, through chromodomain proteins capable of multimerization^18^,^19^, we imagine that HDACs are likely to participate in global feedback reactions.

Long blocks of heterochromatin in plants and metazoans are structurally similar to the heterochromatic domains of *S. pombe* and it is known for some of them that their formation depends on KMT1 methyltransferases. Large H3K9me domains are formed in the olfactory receptor, zinc finger and protocadherin gene clusters during mammalian development, at multiple loci in cells that differentiate or adapt to growth conditions^4^,^5^,^6^,^7^,^13^ and on the inactive X chromosome in mammals (reviewed by Chaligné and Heard^8^). H3K9me is also deposited at transposable elements in the *Drosophila* germline^9^ and, in the very early stages of mouse embryogenesis, as DNA methylation is erased and reprogrammed, the establishment of H3K9 methylation silences retroelements and modulate their ability to control gene activity through regional changes in chromatin structure^10^,^11^,^12^. Although widely different pathways might contribute to the formation of these heterochromatic domains, some basic mechanistic principles relying on the evolutionarily conserved ability of modified nucleosomes to recruit histone-modifying enzymes are likely to be shared. Indeed, modelling developed for *S. pombe* can be applied to other organisms^61^,^62^,^70^. Here we show how transient signals can help establish large heterochromatic domains by facilitating the self-recruitment of histone-modifying enzymes and how the signals can later become dispensable for heterochromatin maintenance. In the case at hand, a strong source was provided by RNAi, but other mechanisms could be substituted for RNAi.

Moreover, our observations document the existence of a Clr4-dependent epigenetic memory in fission yeast heterochromatin. Clearly, wild-type cells do not undergo heterochromatin establishment at each division, indicating the existence of an epigenetic component. The epigenetic mark would be lost in *clr4Δ* cells. The mark could be H3K9me itself or a more complex structure including proteins and RNA molecules associated with the heterochromatic region. Two recent studies have presented evidence that artificially established H3K9me domains can be epigenetically maintained in *S. pombe* in the absence of the initiating sequence-specific recruitment^14^,^15^. In a different system, Polycomb complexes in the early *Drosophila* embryo, it was proposed that histone-modifying enzymes rather than the marks themselves are passed on during DNA replication^71^. In *S. pombe*, interactions between the Clr4 complex and replication factors^69^ might help Clr4 stay associated with chromatin behind the replication fork and/or interactions between Clr4 and protein complexes bound to non-coding RNAs might help maintain heterochromatin as the DNA replication fork passes through.

## Methods

### Strains

*S. pombe* strains were constructed using standard molecular biology and genetics as described in Supplementary Information. Genotypes are shown in Supplementary Table 1. Oligonucleotides are shown in Supplementary Table 2.

### Re-introduction experiments

The *S. pombe* strain PG3437 was used to re-introduce c*lr4*^*+*^ into the *clr4Δ* strains SPK450, SPA26 and PG3404, and for a control cross with the *clr4*^*+*^ strain PG1789, SOM10 was used to re-introduce *clr4*^*+*^ into SOM12 and SOM54, and PG3716 to re-introduce *clr4*^*+*^ into SOM67 and SOM70. Cells were propagated in yeast extract medium (YES), mixed pairwise on SPA plates supplemented with leucin, uracil and adenine, and incubated at 25 °C for 2 days to induce mating and sporulation. The mating-mixes mixes were scraped, resuspended in 1–4 ml 1% glusulase (Roche) in H_2_O, incubated overnight at 33 °C and vortexed for 5 min to obtain preparations of random spores. Random spores were pelleted by centrifugation and washed in distilled H_2_O. For each cross, 90 spores were arrayed on a rich medium plate (YES) by micromanipulation, incubated for 3 days at 33 °C and spore colonies were replica-plated onto selective media to determine the genotypes of the progeny and, when applicable, to assay the expression of *ura4*^+^. Spores destined for fluorescence microscopy were resuspended in EMM2 and incubated for 5 h at 33 °C, at which point prototrophic spores started to germinate. The spores were placed in a home-made growth chamber in EMM2 medium for immediate microscopy or inoculated into 1 ml EMM2 and incubated in a 13-ml culture tube at 33 °C with shaking. Spore germination was synchronous under these conditions. Liquid cultures were imaged at first every ∼2 h and less frequently for late timepoints. Cells were counted with a haemocytometer at each timepoint and an appropriate dilution was made in EMM2 to keep cultures in exponential phase. Cultures were plated on YES plates at the end of each experiment and replica-plated onto selective media to test the effectiveness of the selection.

### Fluorescence microscopy

Cells were imaged with a Nikon Ti Eclipse epifluorescent microscope equipped with a × 100 objective, an Andor Luca camera, a temperature-controlled enclosure and the NIS Elements acquisition programme. The fluorophores were visualized using a Yellow GFP BP HYQ filter set for YFP and a TRITC HYQ filter set for mCherry. Image acquisition times were 500 ms for YFP and 150 ms for mCherry.

### Image Analyses

Fluorescent signals were quantified using a custom-image processing software written in MATLAB. In Figs 2 and 4, the Clr4-independent mCherry signal produced by *ura4:mCherry* was used to outline the boundaries of cell nuclei; average YFP intensities were measured within these boundaries. We found a clear separation in the YFP intensity (around 525, shown in Fig. 2) of silenced and non-silenced cells. This was then used to automatically count silenced and non-silenced cells. In Fig. 5, cell boundaries were identified using *z*-stacked bright-field images and custom-image processing software. The nucleus was found by either mCherry or YFP signal, or, in case where both reporters were silenced, the middle of the cell was taken to be the centre of the nucleus. The initial timepoints, where the automatic cell identification was challenging due to the presence of spores, were quantified by manually defining a point in each nucleus, around which nuclear YFP and mCherry intensities were measured. The results of the automated image-processing software were tested against manually sampled intensities and against choices of silenced versus non-silenced cells made by visual inspection at the intermediate time-point, ∼5 generations. For each sample and at each timepoint, the intensity of at least 1,000 cells was quantified.

## Additional information

**How to cite this article**: Obersriebnig, M. J. *et al*. Nucleation and spreading of a heterochromatic domain in fission yeast. *Nat. Commun.* 7:11518 doi: 10.1038/ncomms11518 (2016).

## Supplementary Material

Supplementary Information Supplementary Figures 1-11, Supplementary Tables 1-2, Supplementary Notes 1-4, Supplementary Methods and Supplementary Reference

Supplementary Movie 1 Establishment of silencing following *clr4*^+^ re-introduction monitored by fluorescence microscopy. The movie shows one time-lapse example with an overlay of the green channel monitoring shut down of GFP in the mating-type region and the red channel monitoring mCherry expression in a euchromatic region. Selected frames are displayed in Figure 3.

Supplementary Movie 2 Same as Supplementary Movie 1 - Green channel.

Supplementary Movie 3 Same as Supplementary Movie 1 - Red channel.
