# Supplementary material for: Nucleation and spreading of a heterochromatic domain in fission yeast
Source: Nat Commun. 2016 May 11;7:11518. doi: 10.1038/ncomms11518 (PMC4865850; doi:10.1038/ncomms11518)
Supplement: Supplementary Information — Supplementary Figures 1-11, Supplementary Tables 1-2, Supplementary Notes 1-4, Supplementary Methods and Supplementary Reference [file ncomms11518-s1.pdf]

Supplementary Figures

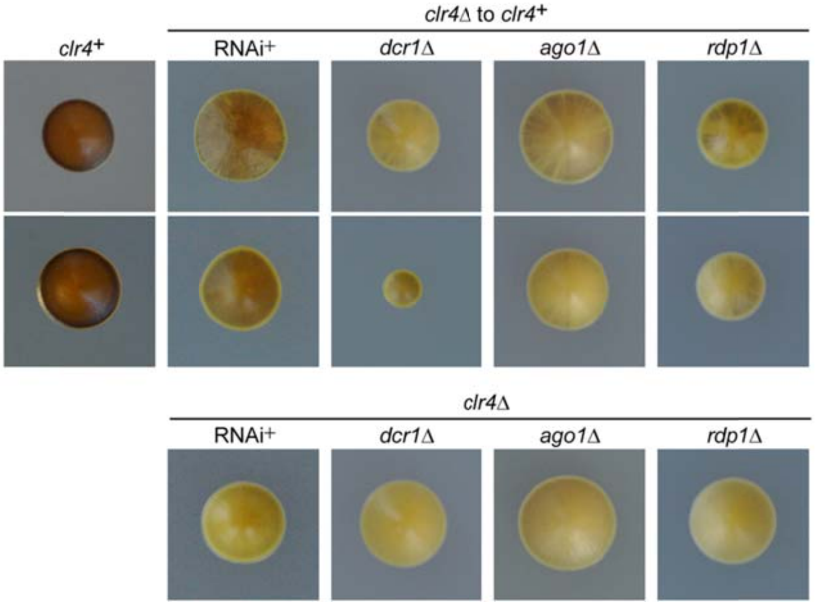

Supplementary Figure 1. Delayed mating-type switching proficiency following re-introduction of *clr4*$^+$. The heterochromatic structure of the *mat2-P – mat3-M* region is necessary for efficient mating-type switching, a gene conversion process in which *mat2-P* and *mat3-M* act as donors of genetic information to convert the expressed *mat1* cassette, producing cells of opposite mating-type. Due to efficient mating-type switching nearly all cells within a wild-type *h*^{90} colony can mate and sporulate, producing colonies that are stained darkly by iodine vapors ('*clr4*$^+$' panels; iodine vapors stain spore walls). Following re-introduction of *clr4*$^+$ through a genetic cross in RNAi proficient (RNAi$^+$) or deficient (*dcr1Δ*, *ago1Δ*, or *rdp1Δ*) backgrounds in which *h*^{90} *clr4Δ::LEU2* cells were mated with *h*$^+$ *his2*$^-$ *clr4*$^+$ strains to substitute *clr4*$^+$ for *clr4Δ::LEU2*, colonies formed by the *h*^{90} *clr4*$^+$ progeny are not stained as darkly by iodine as wild-type *h*^{90} *clr4*$^+$ colonies (compare '*clr4Δ* to *clr4*$^+$' panels above to '*clr4*$^+$' panels). The phenotypic gap, more pronounced in RNAi mutants but also clearly detectable for the RNAi$^+$ strain, is consistent with the delayed silencing of the *ura4*$^+$ reporter shown in Figure 1, also more pronounced in RNAi mutants. These phenotypes reflect slow heterochromatin establishment over the region. The iodine staining of *clr4Δ::LEU2* strains is shown for comparison. Slow establishment was previously reported for RNAi mutants but not tested in an RNAi$^+$ background[1]. The following strains were crossed here: PG3321 and PG3315 (RNAi$^+$); PG4017 and PG3307(*dcr1Δ*); PG4015 and PG3303 (*ago1Δ*); PG4016 and PG4018 (*rdp1Δ*), using SPA supplemented with leucine, uracil, adenine and histidine to set up mating mixes. For each cross, 90 spores were placed on MSA plates supplemented with leucine, uracil and adenine with a micro-manipulator, allowed to form colonies for 4 days at 30°C, stained and photographed, and replica-plated onto selective media including medium lacking leucine to distinguish *clr4* alleles. Some phenotypic variability was observed among the *clr4*$^+$progeny, representative images are shown.

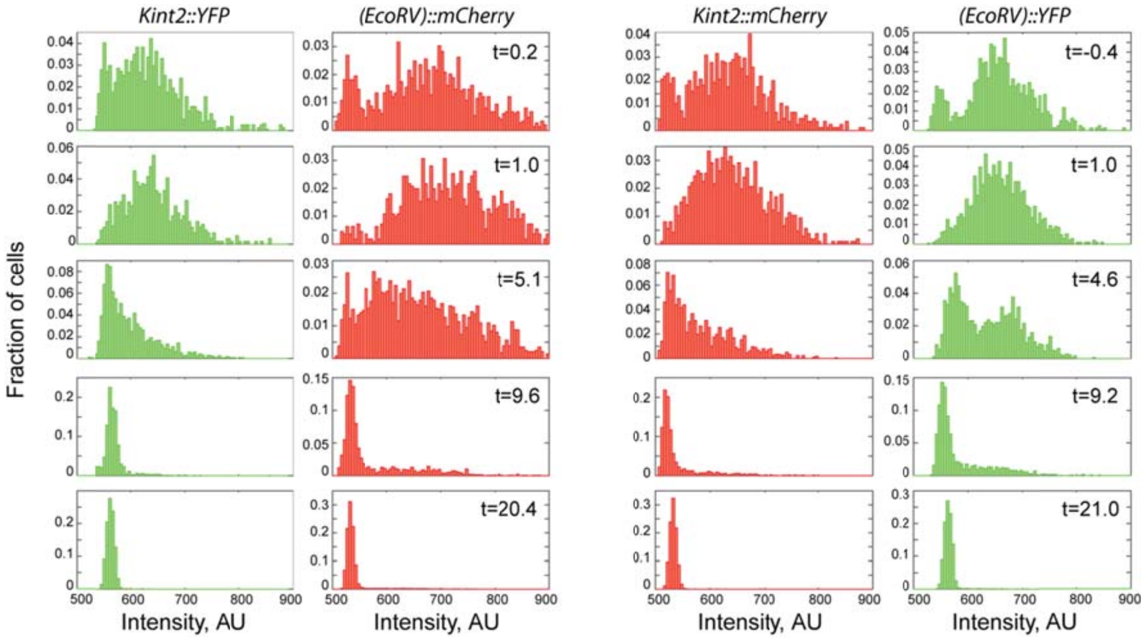

Supplementary Figure 2. Fluorescence distributions in re-establishment experiments. Histograms are shown for selected time-points for the experiments presented in Figure 5. The time t is in generations. Fluorescence intensity increases following spore germination, to a maximum at t=1.0. *Kint2::YFP* and *Kint2::mCherry* are silenced with faster kinetics than (*EcoRV):YFP* and *(EcoRV)::mCherry*.

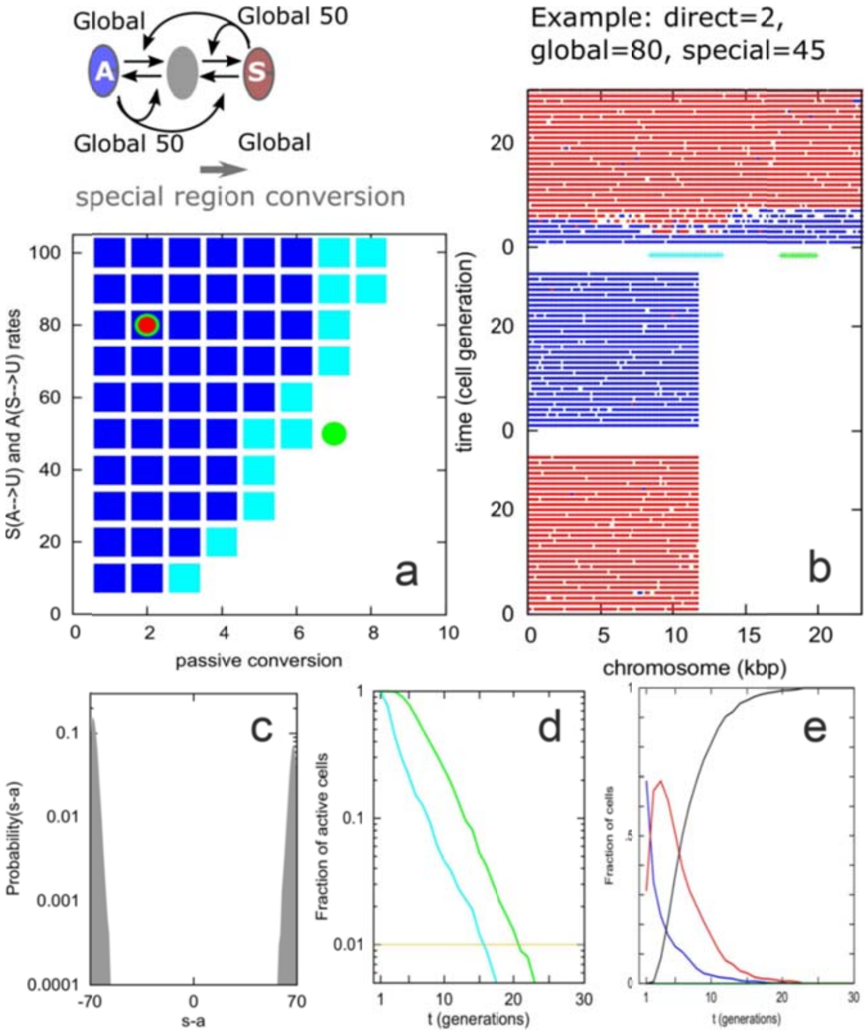

Supplementary Figure 3. Scan of parameters for a three-state model with global recruitment for all nucleosome conversions. Changes in nucleosome states are primarily driven by nucleosomes that are selected at distance x with probability 1/x. The recruitment reactions marked 'Global 50' have a reaction rate of 50 long-range attempts per cell generation. (a) Parameter scan varying the number of recruited and direct reaction attempts per generation. The scan includes a sampling of U to S conversions in the special silencer region. Green circles indicate parameter combinations that reproduce the kinetics of heterochromatin establishment in the full region. Squares indicate parameter combinations where the stability of each epigenetic state following deletion of the special region is > 100 (cyan) or > 500 (blue) simulated generations. Parameter sets reproducing both establishment timing and epigenetic stability are marked by a red circle. One notices that stability is easy to obtain, whereas timing is difficult to obtain. When setting the special region recruitment to A → U instead of U → S, we found no parameters with acceptable timing and acceptable stability. (b-e) Results of simulation for the sole parameter set found to fulfill all criteria, highlighting a marginally acceptable delay between silencing of the two regions (average turn-off time of 3.3 and 7.5 generations respectively, with time for peaking of number of single switched cells at 3.0 generations). This Supplementary Figure illustrates Supplementary Note 3.

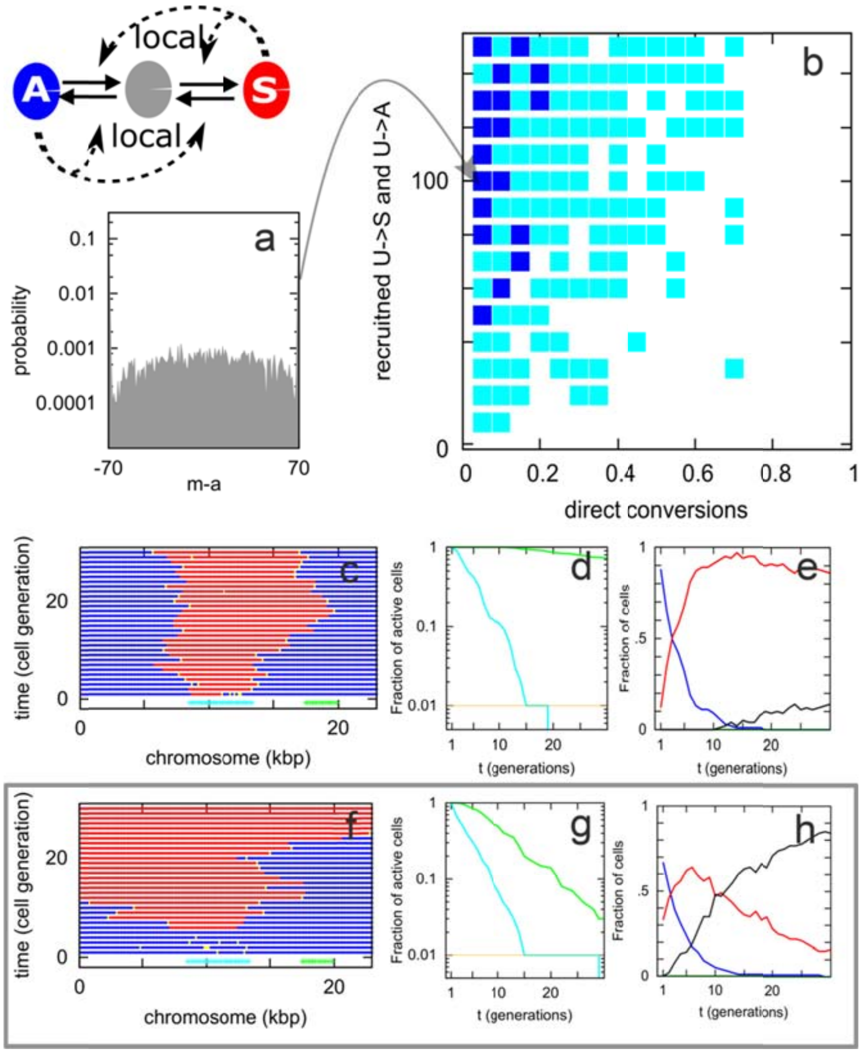

Supplementary Figure 4. Scan of parameters for a three-state model with local recruitment for all nucleosome conversions. (a) illustrates a typical behaviour for the size 70 system (lacking the special silencer region) at a direct conversion rate of $\beta = 0.05$ per reaction type per generation. (b) shows a typical parameter scan, highlighting that some stability can be obtained in principle for very low direct conversion, but also that the timing of reporter silencing in the full region persistently fails. While we vary recruitment rates for reactions away from U state, the two recruitment reactions toward the U state are fixed at 100 attempts per cell generation. (c) shows the dynamics of the system at $\beta = 0.05$ and special region conversion towards silenced state of 22.5/generation, selected to give correct silencing time for the first reporter gene (see panel d). (d,e) show that the second reporter gene is silenced much later than observed experimentally. (f-h) show the dynamics of the system when simulated with recruitment rates of 1000 update attempts per nucleosome per generation, direct recruitments rate $\beta = 0.5$ and special region conversion of A to U of 100 attempts per generation. This Supplementary Figure illustrates Supplementary Note 3. Color coding as in Supplementary Figure 3.

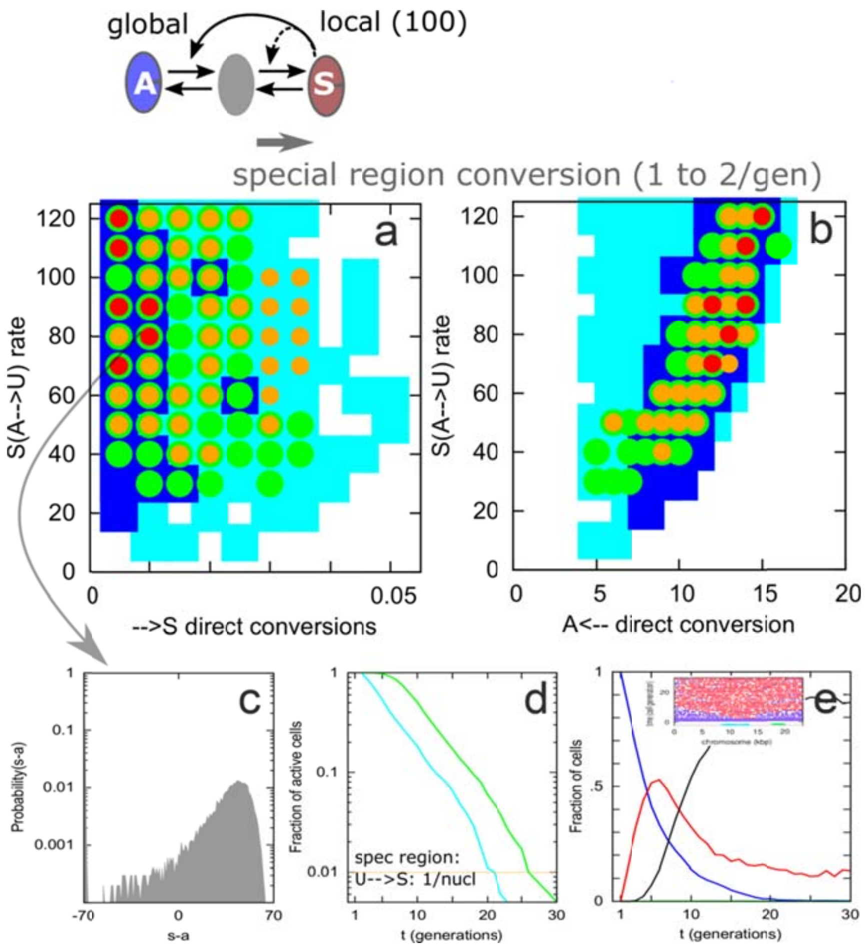

Supplementary Figure 5. Scan of parameters in a three-state model where recruitment occurs only towards the S state, with global S(A → U) and local S(U → S). As marked in the figure, the local recruitment S(U-->S) is fixed at 100 attempts per generation. In (a,b) we also scan variation over the special parameter for direct conversion in the silencer region, giving rise to a number of acceptable fits, however only for the shown extremely low rates of direct conversions towards S. (a) Scan of S(A-->U) versus direct conversions toward S-state, for fixed direct conversion rate toward A sampled as in panel (b). (b) The corresponding direct conversion rates toward A. (c) illustrates that that for this type of motif, the silenced state becomes very noisy, with large cell to cell fluctuations in silencing, which in turn implies that switching of the full region is noisy and thus that there remain a fraction of cells that return to a state with few silenced nucleosomes (e). This Supplementary Figure illustrates Supplementary Note 3. Color coding as in Supplementary Figure 3, with orange circles indicating parameter sets for which the timing of reporter silencing is fulfilled and the stability of chromatin states following deletion of the special region is >100 generations but <500 generations.

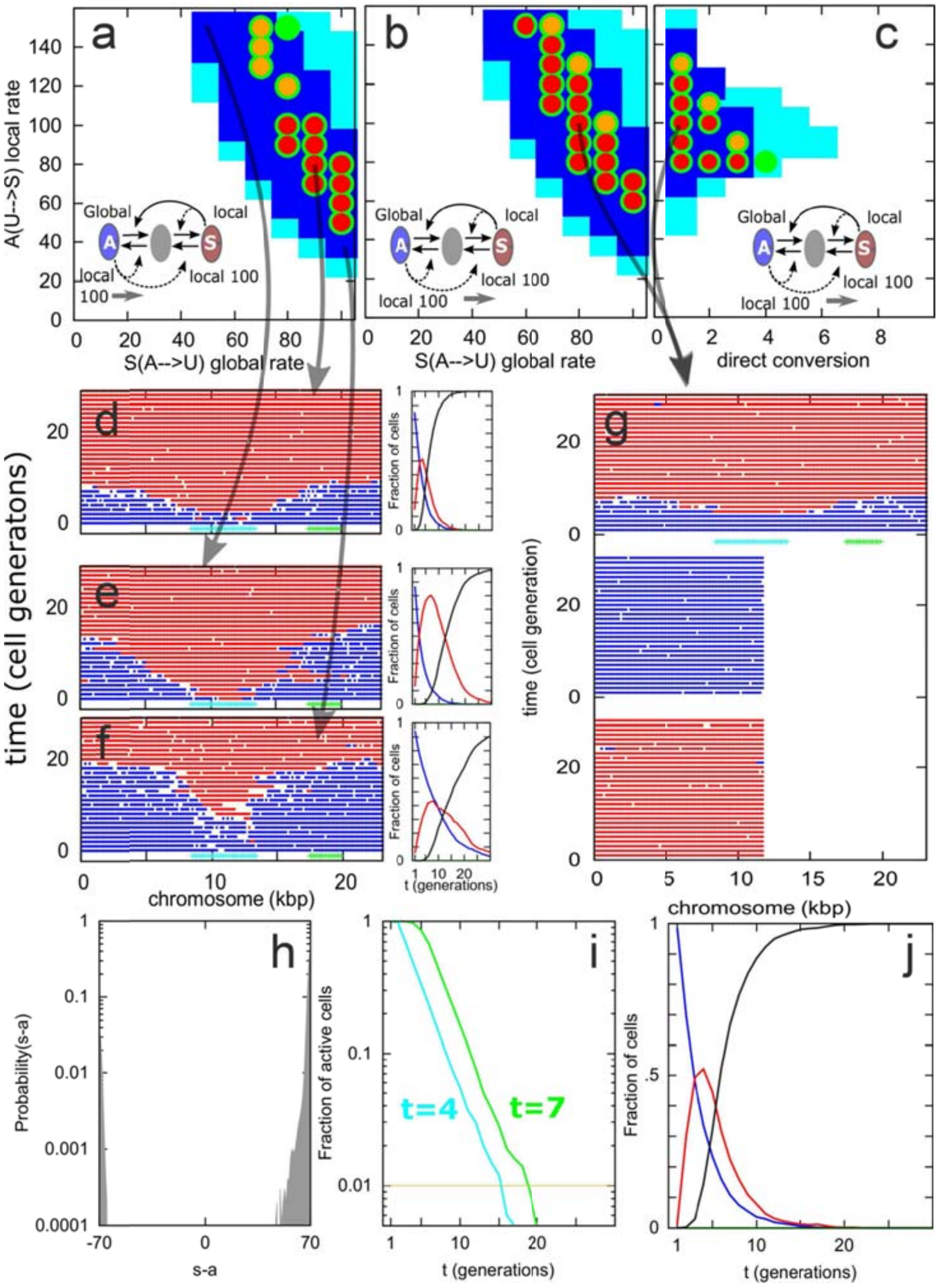

Supplementary Figure 6. Scan of parameters in a standard model with global recruitment from S, that changes an active nucleosome from A to U at distance x with probability 1/x. (a) scans with special region acting on A → U transition (for direct conversion β =1 per generation), whereas (d-f) show examples of behavior for the parameters indicated with grey arrows. The small panels on the right hand-side of (d-f) show the corresponding fraction of cells in each state as function of time. (b,c) scan model with special region acting on U → S transition with (b) simulated for β = 1. (g-j) show the behavior of this model for the parameter sets indicated with arrows from panel (a). This Supplementary Figure illustrates Supplementary Note 3. Color coding as in Supplementary Figure 3 and Supplementary Figure 5.

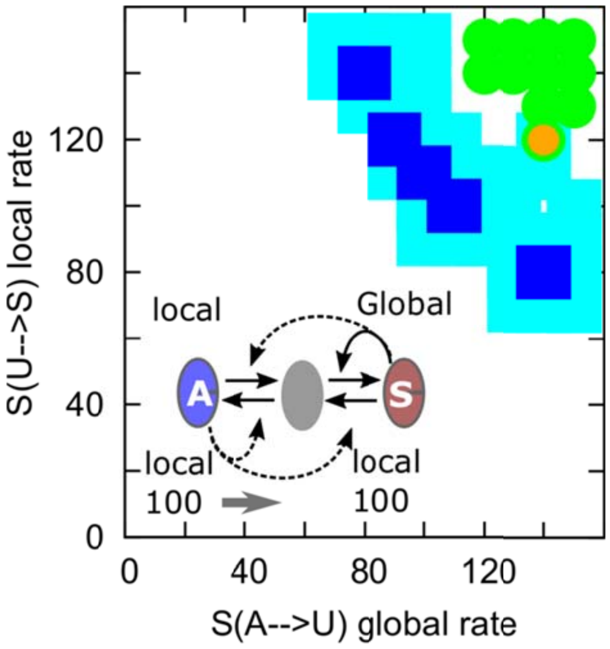

Supplementary Figure 7. Scan of parameters in three-state model, here with global recruitment from S that changes an active nucleosome state U to S at distance x with probability 1/x. Recruitment rates towards active states were set to 100 per generation, and direct conversions to 0.5 nucleosome per generation. The silencer region had increased A → S (marked with gray arrow). In case we instead simulated an increased U → S there was no acceptable timing events (data not shown, as obtained stability anyway is identical to the ones shown). Notice that the correct timing events in upper right corner (green dots) are associated with parameters where the silenced state is much more stable than the active state, and thus the silencing of the second region favored by an overall tendency to become silenced. This Supplementary Figure illustrates Supplementary Note 3. Color coding as in Supplementary Figure 3 and Supplementary Figure 5.

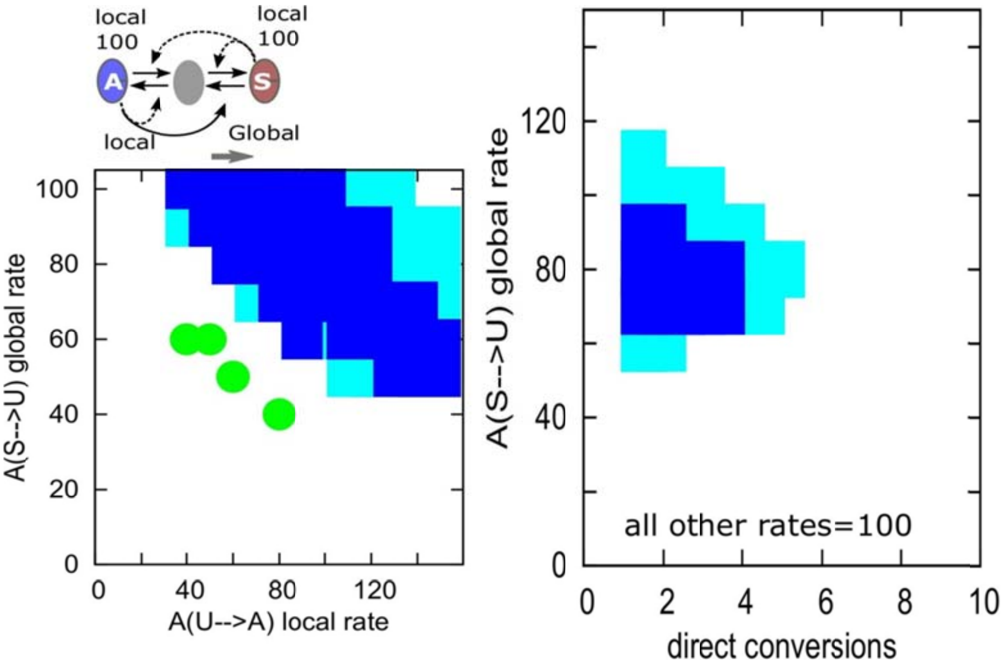

Supplementary Figure 8. Scan of parameters in three-state model, here with global recruitment from A, where recruitments towards S-state are attempted at a rate of 100 per nucleosome per generation. Left panel with direct conversions $\beta = 1$ nucleosome per generation, right panel with $A(U \rightarrow A)$ of 100 per generation (marked with "local 100" on the reaction panel). The above scan also includes a variation over the special parameter for direct conversion in the silencer region. (Notice that in this case, switching of the silencer region provides no direct help to the remaining system, it however reduces the amount of global recruitment towards of U to S. This effect is too weak to give a second switch within the observed time window). This Supplementary Figure illustrates Supplementary Note 3. Color coding as in Supplementary Figure 3.

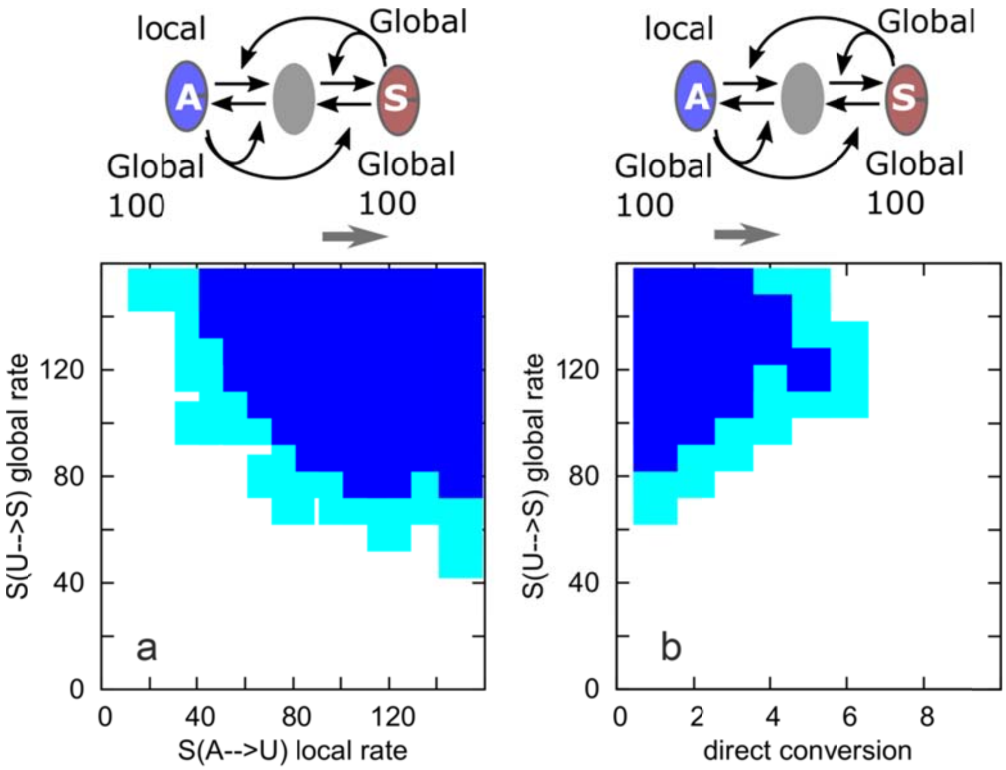

Supplementary Figure 9. Scan of parameters in three-state model, here with one local and three global recruitments. The recruitment attempt rates towards the A state were fixed at 100 as marked on the reaction panel. No parameters gave acceptable timing of the two silencing events. In panel (a), we use direct recruitment rate $\beta = 1$. This Supplementary Figure illustrates Supplementary Note 3. Color coding as in Supplementary Figure 3.

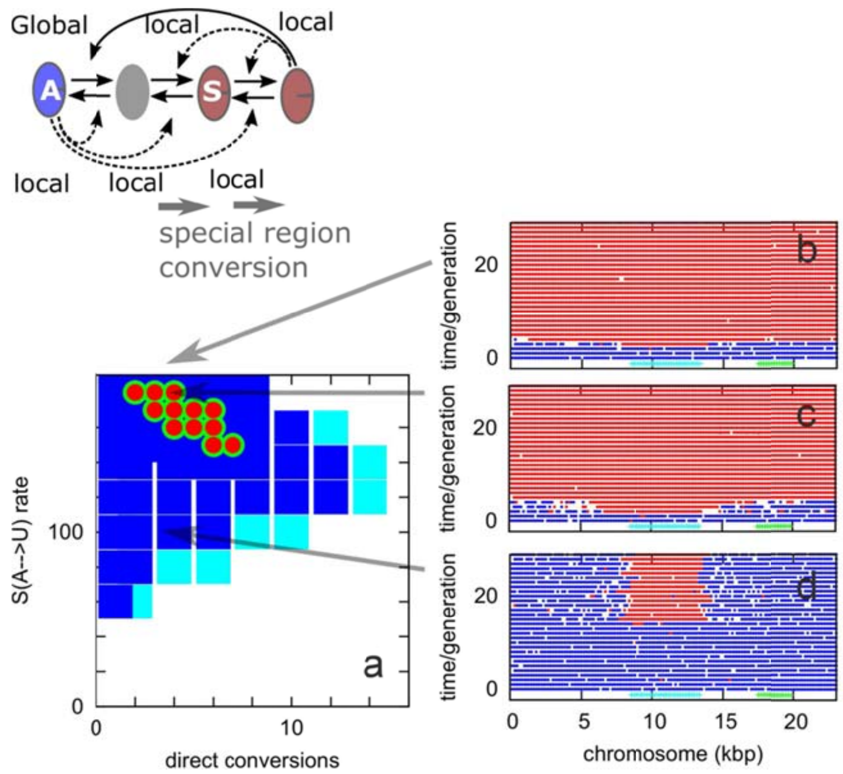

Supplementary Figure 10. Scan of parameters in four-state model, here with one global recruitment and all other recruitments local. All local recruitment reactions are set to the attempt rate of 100 per nucleosomes per cell generation. (b-d) examine one case study for rate of S(A → U) of 200, 180 and 100 global conversion attempts per generations (with special region fixed at a rate of 25 direct conversions for each of the two special reactions). For high global rate one sees that the entire system switches just after the first region is silenced. In contrast, a weaker global rate opens up for a two switch system, where the second region is silenced by a rare switching from a meta-stable state where the first region is silenced, while the rest of the system can remain active. This Supplementary Figure illustrates Supplementary Note 4. Color coding as in Supplementary Figure 3.

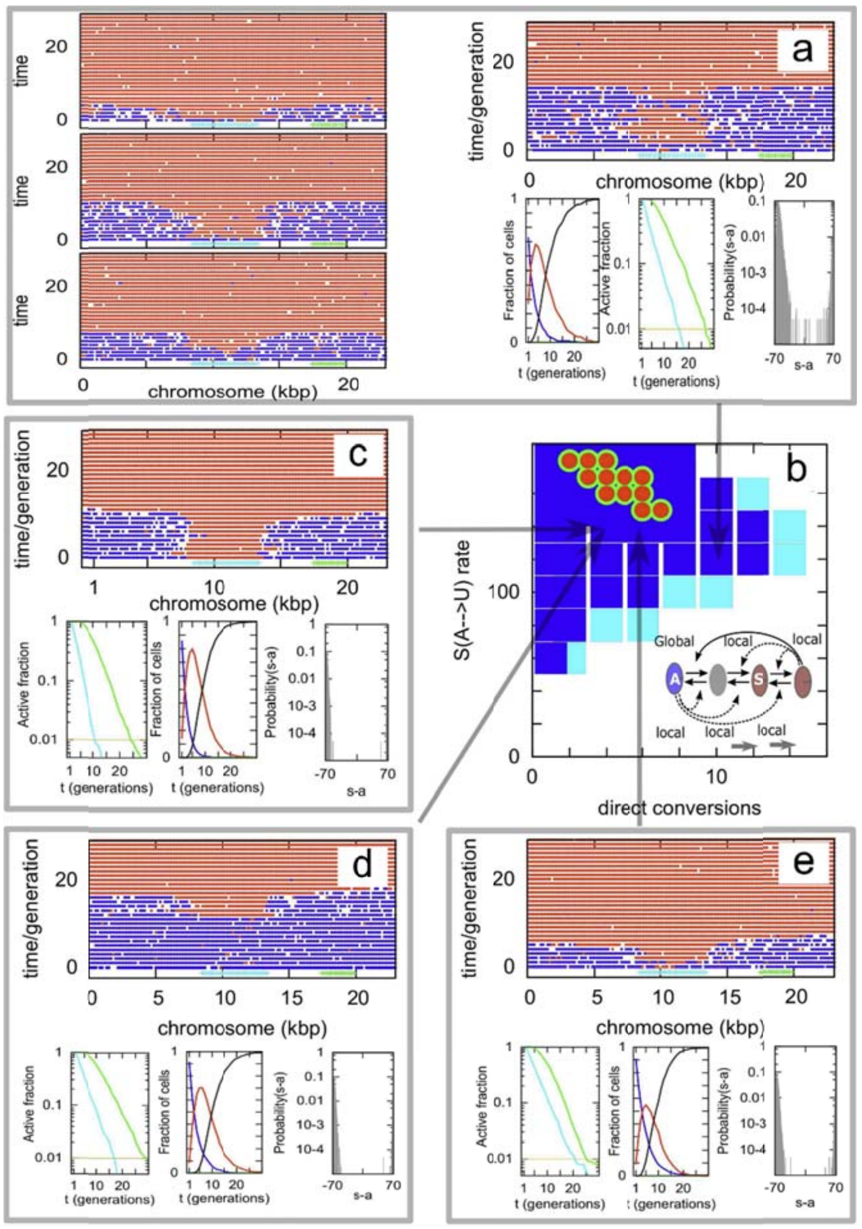

Supplementary Figure 11. Examples of dynamics of four-state model as one varies parameters around the acceptable parameter range. Notice that we select parameters that are not exactly fulfilling our timing constraint. (a) Developments of four cells for the same parameter sets, all using the special region conversion σ = 17.5. The average silencing of first and second gene at four (respectively ten) generations, while the 70 nucleosome region has silenced stability of 2000 and an active state stability of 8000 generations. Notice that the overall behavior approximately matches experimental observations, and does so with different exponential distributions for the two genes. (b) Summary of the parameter scans. Color coding is the same as in Figure 3. (c)-(e) are the same as in (a) except parameter σ: (c) σ = 27.5; (d) σ = 25; e) σ = 20. The figure illustrates 1) that we obtain sensible timing in a window larger than fulfilling our strict criteria, 2) that we can adjust parameters to obtain correct lifetimes of the 70-nucleosome sub-system, and 3) that diverse parameter sets predict different slopes of the exponentially distributed silencing of the two genes. This Supplementary Figure illustrates Supplementary Note 4.

Supplementary Tables

Supplementary Table 1. *S. pombe* strains and their genotypes.

Strain	Genotype
PG1789	mat1-M-smt-0 mat2-P(XbaI)::ura4^{+} leu1-32 ura4-DS/E ade6-216
PG3303	h^{+} his7-366 ago1::kanMX6 leu1-32 ura4-D18 ade6-DN/N
PG3307	h^{+} his7-366 dcr1::kanMX6 leu1-32 ura4-D18 ade6-DN/N
PG3315	h^{+} his7-366 leu1-32 ade6-DN/N
PG3321	h^{90} clr4Δ::LEU2 leu1-32 ura4-D18 ade6-DN/N
PG3404	mat1-M-smt-0 mat2-P(XbaI)::ura4^{+} clr8Δ::kanR leu1-32 ura4-DS/E ade6-216
PG3437	mat1-PΔ17::LEU2 leu1-32 ura4-DS/E ade6-210
PG3700	h^{90} ura4Δ::YFP
PG3716	h^{+} ura4-D18
PG3729	h^{90} (EcoRV)::YFP (SpeI)::ura4^{+} ura4Δ::mCherry
PG3873	h^{90} (XmnI)::ura4^{+} kint2::YFP mat3-M(RV)::mCherry clr4Δ::kanR arg12Δ::natR ura4-D18
PG4015	h^{90} clr4Δ::LEU2 ago1::kanMX6 leu1-32 ura4-D18 ade6-DN/N
PG4016	h^{90} clr4Δ::LEU2 rdp1::kanMX6 leu1-32 ura4-D18 ade6-DN/N
PG4017	h^{90} clr4Δ::LEU2 dcr1::kanMX6 leu1-32 ura4-D18 ade6-DN/N
PG4018	h^{+} his7-366 rdp1::kanMX6 leu1-32 ura4-D18 ade6-DN/N
SOM10	h^{+} ura4Δ::mCherry
SOM12	h^{90} (XmnI)::ura4^{+} mat3-M(EcoRV)::YFP clr4Δ::kanR arg12Δ::natR ura4Δ::mCherry
SOM41	h^{90} kint2::YFP (SpeI)::ura4^{+} ura4Δ::mCherry
SOM54	h^{90} kint2::YFP (SpeI)::ura4^{+} clr4Δ::kanR arg12Δ::natR ura4Δ::mCherry
SOM67	h^{90} (XmnI)::ura4^{+} kint2::YFP mat3-M(RV)::mCherry clr4Δ::kanR arg12Δ::natR ade6-216 ura4-D18
SOM70	h^{90} (XmnI)::ura4^{+} kint2::mCherry mat3-M(RV)::YFP clr4Δ::kanR arg12Δ::natR ura4-D18
SOM75	h^{90} (XmnI)::ura4^{+} kint2::mCherry mat3-M(RV)::YFP ura4-D18
SOM81	h^{90} (XmnI)::ura4^{+} kint2::YFP mat3-M(RV)::mCherry ura4-D18
SPA26	mat1-M-smt-0 mat2-P(XbaI)::ura4^{+} clr7Δ::kanR leu1-32 ura4-DS/E ade6-216
SPK450	mat1-M-smt-0 mat2-P(XbaI)::ura4^{+} clr4Δ::kanR leu1-32 ura4-DS/E ade6-210

Supplementary Table 2. Oligonucleotides used in strain constructions.

Oligonucleotide	Sequence
GTO-357	GAAGATCTCATATGAACTTTGCTTTTAAACCTTTAATTTCGATCC
GTO-358	GACCATGGGCATGCTAAAAAAAGACTAATGTAAAATTTTTTTGGTTGG
GTO-359	GAAGATCTCATATGATTCCCGAGCCTCCAAAAAAGAAGAGAAAGGTCGAA<br>TTGGTGAGCAAGGGCGAGGAGCTG
GTO-360	GACCATGGGCATGCCTTGTACAGCTCGTCCATGCCGAG
GTO-414	CAGATGTGTGTAAAATTTCCATCGTTTATCTATAAAGATGGTTTATTGAT<br>GAGAAAGATAGTAAAAAATAGTGTCGGCTATATTATTACATCAGTGCG<br>CTATCAGTTTAAAAGGTTGGGCCTACTAACTTAACATATACTACACCTC<br>AAGAAAAAGAAAGAAATGACCACTCTTGACGACACGG
GTO-415	GCAACAAAAATATTCAATTGACTTATCAGTGATATGAACGGAGTAATTCT<br>ATGAAATGTGAATATGAAAAGGAAGCCATCTCAAATAATACTTCTTATTT<br>GCACAGCAGAAATTTGATTATTGCTTGGCTCAAGTACATGGTGAGTATGA<br>CATTATTATTGAGAACGACCTGGCATCTAGGGGCAGGGCATGCTCATG
GTO-428	GACATATGATTCCCGAGCCTCCAAAAAAGAAGAGAAAGGTCGAATTGGTG<br>AGCAAGGGCGAGGAGGATAAC
GTO-429	GAGCATGCTTACTTGTACAGCTCGTCCATGCCGCCGGTG

Supplementary Note 1

Varying Models and Parameters

This Supplementary Note and Supplementary Notes 2-4 probe models and parameters with the aim of pinpointing the minimum requirements and most robust ways of reproducing the observed kinetics of heterochromatin establishment and maintenance. Importantly, several models could potentially fit the data. We discuss model quality in terms of the fraction of parameter sets that provide sensible fits to the data. We already know from earlier modelling that epigenetic bi-stability requires at least one of the read-write recruitment reactions to be global[2]. From experiments in the present study we further know that switching chromatin state in one region is substantially delayed compared to switching in another region that is separated by about 40-50 nucleosome units along the chromosome. As we will see, this delay constrains the acceptable models more than the simple requirement for bi-stability. In particular, robust fitting requires that some recruitment reactions act only on nearest neighbors. In the following, these nearest neighbor interactions are denoted local recruitment processes.

Throughout the analysis we maintain some standard parameters fixed. These include a basic frequency of rate of recruitment of non-shown reactions of 100 attempts per nucleosome per generation. Compared to our original model[2], this attempt rate exceeds the minimum of ten update attempts per generation needed for robust behavior. However, the models presented here use an event-based updating that is designed so we first chose a specific reaction, then a pair of nucleosomes. The update only takes place if the chosen nucleosomes are of a type allowing the reaction. Because there are more distinct processes here than in earlier models, more of the attempted reactions do not take place. In our typical simulations with about 100 attempts per reaction type per generation, only about one to five recruitment events per nucleosome are successfully completed in each generation (i.e. this balances the effects of cell division and the direct conversions that are antagonistic to the current epigenetic state).

The simulated systems are respectively the full mating-type region including nucleosomes from positions 1 to position 140 and a mutant system. The full system contains a special region from position 51 to position 80, to which one additional direct conversion is assigned. The two reporter genes are respectively in the region [51, 80] and in the region [105, 119]. The mutant system consists of the above system, except that 70 nucleosomes including the region [50, 80] were removed, leaving a sub-system of 70 nucleosomes that experimentally was found to be bi-stable with a ~ 2000 generation stability in each epigenetic state[3].

Algorithm:

The model allocates a direct conversion rate β to a number of reactions between possible states i and j of a nucleosome, $R(i \rightarrow j)$. In addition, the special region is assigned one direct conversion rate σ towards the silenced state. The model also has preassigned rates for recruited conversions $R(i \xrightarrow{k} j)$ where conversion of i to j requires that another chosen nucleosome is in state k. In some cases this other nucleosome is chosen locally (nearest neighbor) to

the nucleosome in question. For other reactions it is associated to a long-range read-write enzyme implying that the recruiting nucleosome is chosen globally at a distance x proportional to 1/x.

The model is executed as an event-driven Monte-Carlo simulation (simplified Gillespie algorithm), where one at each step assigns each possible reaction a possible reaction time $t_R = -ln(ran)/rate(R)$ and subsequently choses the reaction with the shortest time t_s for an attempt. The attempt consists in selecting a nucleosome for the chosen change type and in performing the change if the nucleosome state is the one required for the considered reaction. In case the chosen reaction is a recruitment attempt, one also selects another nucleosome, and then only performs the change if the state of the nucleosome is consistent with the chosen reaction. In case one of these requirements fails, no reaction takes place.

For each attempt, the time is updated by adding t_s to the time. Subsequently the above step is repeated. When the time of one generation is reached, a cell generation event takes place. In this event each nucleosome in the system is replaced by a U nucleosome with probability 1/2.

From the above, one can infer that many attempted reactions are aborted. We selected the above procedure because one of the reactions was a global recruitment reaction where the recruiting nucleosome was chosen at distance x with probability 1/x. This made it less elegant to use a more efficient Gillespie simulation where updating times was adjusted to available concentrations and each attempt subsequently led to a conversion.

Supplementary Note 2

Criteria for accepting a parameter set as a fit to data

The main constraint is bi-stability of the smaller system of 70 nucleosomes, obtained by removing 70 nucleosomes that include the 30 nucleosome region with more favored silencing directed processes[2]. The experiments constrain the models by requiring a bi-stability with an average lifetime for each epigenetic state of ~ 2000 generations. In Supplementary Figures 3-11, we use blue squares to mark parameters where each epigenetic state survives more than 500 simulated generations and cyan squares to mark parameters where this constraint is relaxed to 100 generations. Notice that in the parameter scan we stop the simulations after 500 generations, and do not calculate the average lifetime of each state, and thus also accept parameters that give higher stability than observed in experiments. The philosophy is that the precise behavior of the real system only can be reproduced for a very narrow set of parameters, but the qualitative epigenetic behavior should be robust.

The additional constraint imposed by the experiments presented in the current study is the timing of the silencing of the two reporters. The reporter inside the special silencer region is turned off within a characteristic time t_1 of about 3.2 (YFP) to 3.5 (mCherry) generations whereas the more distant promoter is turned off with an average lifetime t_2 between 6.3 (YFP) and 7.7 (mCherry) generations. When scanning parameters to fit these turn-off rates, we allow

some uncertainty and accept turn-off time of $t_1 \in [2, 5]$, respectively $t_2 \in [t_1+2, 8]$ (in unit of cell generations). Thus we require that the reporter inside the special region is turned off within 2 to 5 generations, that the reporter outside this region is turned off at least 2 generations after the first reporter, but also that its average turn-off time is less than 8 generations. We also require that the ratio of cells where one reporter is on and the other off never exceeds 70% (In experiments the maximum of this number was between 30% and 50%, and was quite sensitive to experimental thresholds on detected luminosity). Finally we demand that this maximum occurs in the interval [2.5, 6] generations (in experiments this maximum was at about 4 generations). When these timing criteria are fulfilled we mark the chosen parameter set with a green circle. Noticeably, in all cases one must select a threshold of methylation at which a promoter is considered to be repressed. This we arbitrarily put to a level of methylated minus acetylated nucleosomes to be 8 of the 30 nucleosomes in the first region, and 8 out of the 14 nucleosomes in the second region. This threshold is in general not important for the result, except in the case where there is no recruitment in one direction.

The fulfillment of one criterion does not guarantee fulfillment of the other. In Supplementary Figures 3-11, we mark parameter sets where the requirements for epigenetic stability and for the timing of establishment are both fulfilled with a red circle. If epigenetic stability is only fulfilled with a 100 generation limit and timing fulfilled we mark the parameter set with an orange circle. In addition to scanning for the parameters displayed in the plots we always scan over a range of not shown parameters, with the result that for some sets of shown parameters conditions that fulfill the timing requirement and conditions that fulfill the stability requirement are both found, yet no conditions that simultaneously fulfill both criteria. In particular, we persistently scan over the parameters that quantify the extra silencing in the special region. This extra silencing is always assumed to be of the form of direct conversions towards the silenced state.

Supplementary Note 3

Three-state models and their ability to fit the data

Traditional model with global recruitment for all transitions

Supplementary Figure 3 shows that one can fit the obtained data with the standard model[2], with the addition of a 1/x distance dependence for the read-write recruitment reactions. The bi-stability is easy to obtain, also for substantial levels of direct conversions, whereas obtaining a difference in timing for the silencing of the two reporter genes is a highly fragile feature. Differential timing requires a very active conversion towards the silenced state in the silencer region. In particular, it is easy to fine tune the silencing time of the first promoter, but very difficult to obtain a substantial delay until the second promoter is turned off. This fragility inspired us to seek other ways of obtaining the experimentally observed timing.

Model with purely local recruitments

When assuming that all recruitments are limited to nearest neighbors, we cannot obtain true bi-stability. However, for very low rates of direct conversions, one can obtain moderately long lasting states (Supplementary Figure 4). Thus, one can obtain a ~ 500 generations stability for a symmetric system of size 70 when assuming that there are fewer than 0.15 direct conversions for each of the reactions in a three-state system (using between 10 and 150 direct recruitment attempts for each reaction per generation). As a hallmark of this fragility of inherited states, Supplementary Figure 4a shows that all values of methylation of the system are equally likely, except the extreme values. This reflects a system which has a stability of extreme states of order $1/\beta$ and thus is very fragile to direct conversions.

The fragility of the system is related to its intrinsic dynamics, where the boundary between the silenced and the active region performs a random walk (Supplementary Figure 4c). That is, assuming that the subsystem of 70 nucleosomes is balanced between silenced and active conversions, then the full system is also exposed to a balanced random walk outside the specially silenced region. This implies 1) that much more activity is required to obtain a correct timing for the silencing of the second promoter, a feature that is explored in Supplementary Figure 4f-h. Further, it implies that silencing of the second promoter is quite often lost (for about a third of the cells times), before the entire region is silenced. Overall, the phenomenology of the model with only local recruitments is inadequate both by its absence of bi-stability and by the lack of persistent silencing of an already silenced second promoter.

Recruitment in only one direction

Supplementary Figure 5 shows that if recruitment is limited to one direction then it is difficult to obtain parameters that are consistent with our experimental constraints. It is possible to obtain bi-stability, but only with a very small direct conversion rate towards the S-state. With this very low rate it is possible to obtain correct timing.

Model with global recruitment from A to U

Supplementary Figure 6 shows that the standard model indeed can give acceptable behavior over a moderate range of parameters (Figure 6a-c), irrespective of whether the special region is assigned an increased deacetylation (6a), or an increased methylation rate (6b-c). One sees that the model can work with the special region modifying either reaction towards the silenced state, with the overall tendency that in order to fit the measured timing the special region activity on the A → U choice needs to be about half of the similar fits using U → S reaction. Supplementary Figure 6d represents an acceptable timing (using special conversions A → U of 13.5 attempts per generation), whereas Supplementary Figure 6e and 6f illustrate two ways in which the model may fail to fit the observed timing of the two consecutive silencing events: In Supplementary Figure 6e the second silencing comes too late, which in turn gives too large an interval where one region is silenced and the other still active. In Supplementary Figure 6f the first silencing comes too late.

Supplementary Figure 6g illustrates an acceptable fit to the observed data, using a version of the model where the special region acts by direct conversions U → S at a rate of about 30 attempts per generation. The overall behavior of the model is further examined in the rest of the figure. Supplementary Figure 6h shows that the system is tilted towards the repressed state, and thus does not fit the approximately equal stability of the alternative epigenetic states in the 70 nucleosome mutant system[3]. Supplementary Figure 6i illustrates that this parameter set gives a simple spreading type delay of ~ 3 generations between silencing of the two reporters, whereas Supplementary Figure 6j illustrates that the chosen parameter set reproduces the experimentally observed times for both silencing events. In the main text we show an example of a working parameter set for this model, which is both balanced, displaying correct epigenetic stability, and able to reproduce the observed timing. The example used in the text uses a direct conversion rate of 2.5 nucleosomes per generation which is more noisy than the example used in Supplementary Figure 6d-j.

Model with global recruitment from U to S

Supplementary Figure 7 shows that the model with global recruitment acting from S in S(U → S) in principle can provide bi-stable behavior, but only when the rate β of direct conversions is low (the scan in the figure used β = 0.5). Therefore, in order to get 70-nucleosome systems bi-stable, this motif needs a direct conversion rate substantially lower than the β = 1 conversion per nucleosome per generation that was standard in Supplementary Figure 6.

Apart from the overall fragility of bi-stability, the main objection to the model is that it persistently predicts too long a time for silencing of the second reporter. While the characteristic time of reporter silencing in the special region can be tuned by adjusting the direct transition rate in the special region, this silencing does not provide sufficient additional repressive modifications in the remaining system. The global impact of the long ranging S(U → S) from the primary silenced reaction is just too weak.

Model with global recruitment towards A

Models with a single global recruitment acting towards the A state would have the same stability for the 70 nucleosome system as the symmetric version of the above models. Given that one only obtained acceptable stability when global recruitment acted on the modification reaction that was furthest away from the recruiting state, we can restrict our investigation to the case of global recruitment A(S → U) and assume local recruitment A(U → A). In Supplementary Figure 8 one sees that we fail to find parameters that allow us to reproduce the experimental observations.

The model easily provides acceptable bi-stable behavior, but there are no parameters with acceptable ability to switch off the second reporter. The main difficulty here is that a switched sub-region only influences its surroundings very little. Thus silencing of the second reporter persistently takes too long.

When the special region is silenced, the rest of the system is exposed to fewer globally acting recruitment reactions. However, the rest of the system is similar to the 70-nucleosome system that was required to be strongly bi-stable without the special silenced region. The stability of the active region is weakened because of the separation of the 70-nucleosome system into two parts that act more weakly onto each other than if they were neighbors. This is however not enough to allow us to reproduce the timing of the observed silencing of the second reporter.

Model with all global except one local recruitment

The above analysis points to S(A → U) as the only recruitment reaction that by being long-range can allow all other recruitment reactions to be local. To examine whether this reaction in fact needs to be global, we now consider the model in which S(A → U) is local, and the other reactions are all global. As we see from Supplementary Figure 9 such models easily reproduce bi-stability (as expected), but we also see that correct timing cannot be obtained in the absence of long range de-acetylation recruitment. In fact, again, the difference between the system with the special region and the mutant system without this region becomes too small to allow substantial difference in stability of the large and the small system.

Overall, we persistently fail to obtain any working motif in the absence of long-range recruited de-acetylation.

Summary of three-state models

Overall we find that among three-state models the recruited de-acetylation S(A → U) needs to be long-range. Adding other long-range recruitment reactions increases the robustness of epigenetic states to direct modifications, but at the same time makes it more difficult to obtain the observed delay of silencing between the two spatially separated genes. Further, we find that three-state models only provide acceptable robustness toward spontaneous direct conversions when the de-methylation reactions are also directed by recruiting read-write enzymes.

We conclude that the motif in Figure 6 was the only three-state motif that reproduced both the epigenetic bi-stability of the *ΔK* mutants and the observed delay in spreading of the silenced mark.

Supplementary Note 4

Four-state models and their ability to fit data

We examined some four-state models with respect to their ability to provide bi-stability, and, respectively, fit the observed timing. Due to the large variety of options for how the six conversion reactions can be catalyzed, we have however limited ourselves to a simple extension of the model in Figure 6 and Supplementary Figure 6. In this extended model, shown in Supplementary Figure 10, we assume that only the reactions S*(A → U) is global, whereas all other conversion reactions are exposed to locally acting read-write enzymes (S* denotes the extreme state on the silenced side of the conversion reactions). Overall we also find here that

absence of recruitment towards the active state limits the possibility for direct conversion rates to unrealistically small values (in analogy with Supplementary Figure 5, but data not displayed here). However, allowing all reactions to be recruited opens for a qualitatively new way of obtaining a substantial difference in silencing times, as seen in Supplementary Figure 10.

Supplementary Figure 10 examines a quite promising four-state model, demonstrating that adding one additional step along the silencing pathway provides substantial additional robustness to the stability against direct conversions. Also, one obtains a substantial regime of parameters with acceptable timing, parameters where the single global recruitment is 150-180 attempts per generation and where the special region conversions occur at a rate of 20-25 per generation for both the reactions $U \rightarrow S$ and $S \rightarrow S^*$. Noticeably, the correct timing also gives bounds on the direct conversions, here to be above 3 and below 8 conversion attempts per nucleosome per generation. To balance these stability-reducing conversions this in turns means that the four-step model can reproduce results with recruited conversions of up to at least 8 actual changes per generation. The four-step model allows for many active changes in nucleosome states.

For smaller global recruitment rates, silencing of the second reporter is delayed compared to the experiment. For smaller direct conversions, one needs a small special conversion to match the first silencing, which in turn also delays the second silencing. Supplementary Figure 11 examines silencing dynamics of the four-state model for different parameter values. Supplementary Figure 11a illustrates four examples of the spatio-temporal development towards silencing for a parameter set that would be close to consistent with the experimental data. This set of parameters also predicts different exponential slopes for the silencing of the first (cyan) and second gene (green) respectively.

Overall, considering more than three states, and thus three steps for changing a nucleosome in the active state to a nucleosome in the silenced state, indeed makes bi-stability much more robust to the overall dynamics of nucleosome modifications as well as to their active replacements by external factors. Also, it becomes easier to obtain parameters that give a substantial time-delay between the first and the second silencing event.

Importantly, there is a substantial range of parameters where the first region is silenced as a bi-stable system with a relatively short lifetime, whereas the second region is silenced as a second bi-stable system with an exponentially distributed lifetime that is substantially longer. This two-step switch contrasts with the conventional/alternative spreading scenario that results in a close-to-constant time interval between the first and the second silencing event. Instead the double switch behavior reflects two stochastic events, with the silencing of the first gene acting as a 'fuse' that does not spread linearly to the second gene, but rather makes the second silencing event more likely. In this scenario, the second silencing sometimes happens 1 generation after the first silencing, but also sometimes the second silencing instead happens 10 or more generations later.

Supplementary Methods

Construction of strains with fluorescent reporters. The plasmid pGT188 (Thon *et al.*, 2002) contains the *ura4*$^+$ gene and its control regions in a 1.8 kb *Hin*dIII fragment of genomic DNA. The 785 bp *ura4*$^+$ ORF in pGT188 was replaced with the YFP ORF fused to an N-terminal SV40 NLS, introducing an *Nde*I site at the NLS initiating methionine and an *Sph*I restriction after the YFP stop codon (pEP1 plasmid). For this cloning, YFP was amplified with GTO-359 and GTO-360 on pDUALYFH1c[4], the PCR product was digested with *Nde*I and *Sph*I and ligated to pGT188 amplified with GTO-357 and GTO-358 and digested with *Nde*I and *Sph*I. The mCherry ORF was substituted for YFP in pEP1 between the *Nde*I and *Sph*I restriction sites to make pOM6. For this cloning, the mCherry ORF was amplified with GTO-428 and GTO-429 on pML40. The *Hin*dIII inserts of pEP1 and pOM6 were used to replace the *ura4*$^+$ ORF at the endogenous *S. pombe ura4*$^+$ locus, producing *ura4::YFP* and *ura4::mCherry* strains. pEP1 and pOM6 were also used to replace *ura4*$^+$ at other loci where the 1.8 kb *ura4*$^+$ *Hin*dIII fragment had previously been integrated, producing *(EcoRV)::YFP* and *(EcoRV)::mCherry* by replacement of *(EcoRV)::ura4*$^+$ (ref. 5); *Kint2::YFP* and *Kint2::mCherry* by replacement of *Kint2::ura4*$^+$ (ref. 6); and *(XbaI)::YFP* by replacement of *(XbaI)::ura4*$^+$ (ref. 7). For the replacement of *(XbaI)::ura4*$^+$, YFP was first cloned into pAK67[7] by swapping *AvrII-BlpI* restriction fragments between pEP1 and pAK67, producing plasmid pOM3 which was digested with *Hin*dIII prior to transformation. Chromosomal integrations were obtained in *swi6-115* or *clr4Δ* cells.

Mating-type regions with two fluorescent reporters were constructed stepwise by first integrating *ura4*$^+$ in the mating-type region of strains with one fluorescent reporter. pGT234[3] digested with *Sac*I-*Kpn*I was used to introduce *(XmnI)::ura4*$^+$ centromere-proximal to *IR-L* in respectively *mat3-M(RV)::mCherry* and *mat3-M(RV)::YFP* strains and pGT229 (Thon and Friis, 1997) digested with *Xba*I-*Pst*I was used to introduce *(SpeI)::ura4*$^+$ centromere-distal to *IR-R* in respectively *Kint2::YFP* and *Kint2::mCherry* cells. *(XmnI)::ura4*$^+$ *IR-L mat3-M(RV)::mCherry* cells were crossed with *Kint2::YFP IR-R (SpeI)::ura4*$^+$ cells, and *Kint2::YFP mat3-M(RV)::mCherry* recombinants were obtained in the FOA-resistant progeny. *Kint2::mCherry mat3-M(RV)::YFP* recombinants were obtained in the same way. Finally, mating-type regions with fluorescent reporters were tagged by integrating again *(XmnI)::ura4*$^+$ (pGT234) centromere-proximal to *IR-L* or *(SpeI)::ura4*$^+$ (pGT229) centromere-distal to *IR-R* to permit selection of the mating-type region of interest in the progeny of crosses. *(XmnI)::ura4*$^+$ and *(SpeI)::ura4*$^+$ are respectively 451 bp and 416 bp from the heterochromatic domain, their expression is independent of Clr4[3].

Chromosomal insertions and the integrity of the mating-type region were tested by Southern blots at each step of the strain constructions.

Construction of selectable *clr4*$^+$ allele for re-introduction experiments. The *arg12*$^+$ ORF was replaced with the *nat* gene[8] using a PCR product made with long primers providing ho-

mology with the *arg12*$^+$ locus (GTO-414 and GTO-415) to create the *arg12Δ::natMX6* allele near *clr4Δ*. The *arg12*$^+$ and *clr4*$^+$ loci are at 5.5 kb from each other.

Supplementary References

1. Hall, I. M. *et al.* Establishment and maintenance of a heterochromatin domain. *Science* **297**, 2232-2237 (2002).
2. Dodd, I. B., Micheelsen, M. A., Sneppen, K. & Thon, G. Theoretical analysis of epigenetic cell memory by nucleosome modification. *Cell* **129**, 813-822 (2007).
3. Thon, G. & Friis, T. Epigenetic inheritance of transcriptional silencing and switching competence in fission yeast. *Genetics* **145**, 685-696 (1997).
4. Matsuyama, A., Arai, R., Yashiroda, Y., Shirai, A., Kamata, A., Sekido, S., Kobayashi, Y., Hashimoto, A., Hamamoto, M., Hiraoka *et al.* ORFeome cloning and global analysis of protein localization in the fission yeast *Schizosaccharomyces pombe*. *Nat Biotechnol.* **24**, 841-847 (2006).
5. Thon, G. & Klar, A. J. The clr1 locus regulates the expression of the cryptic mating-type loci of fission yeast. *Genetics* **131**, 287-296 (1992)
6. Grewal, S. I. & Klar, A. J. A recombinationally repressed region between mat2 and mat3 loci shares homology to centromeric repeats and regulates directionality of mating-type switching in fission yeast. *Genetics* **146**, 1221-1238 (1997).
7. Thon, G., Cohen, A. & Klar, A. J. Three additional linkage groups that repress transcription and meiotic recombination in the mating-type region of Schizosaccharomyces pombe. *Genetics* **138**, 29-38 (1994).
8. Sato, M., Dhut, S., Toda, T. New drug-resistant cassettes for gene disruption and epitope tagging in *Schizosaccharomyces pombe*. *Yeast* **22**, 583-591 (2005).
